# Ophidiomycosis surveillance of snakes in Georgia, USA reveals new host species and taxonomic associations with disease

**DOI:** 10.1038/s41598-020-67800-1

**Published:** 2020-07-02

**Authors:** Ellen Haynes, Houston C. Chandler, Benjamin S. Stegenga, Laura Adamovicz, Emilie Ospina, Dessireé Zerpa-Catanho, Dirk J. Stevenson, Matthew C. Allender

**Affiliations:** 1 Wildlife Epidemiology Laboratory, College of Veterinary Medicine, University of Illinois at Urbana-Champaign, Urbana, IL USA; 2 The Orianne Society, Tiger, GA USA; 3 Department of Plant Biology, University of Illinois Urbana-Champaign, Urbana, IL USA; 4 Altamaha Environmental Consulting, Hinesville, GA USA

**Keywords:** Fungal infection, Herpetology

## Abstract

Ophidiomycosis (snake fungal disease) is caused by the fungus *Ophidiomyces ophiodiicola* and threatens snake health worldwide. It has been documented throughout the eastern United States and severe cases have recently been reported in Georgia, USA. To evaluate disease distribution and prevalence in this state, 786 free-ranging snakes were examined for skin lesions consistent with ophidiomycosis and swabbed to detect *O. ophiodiicola* DNA using qPCR. Sampled snakes represented 34 species and 4 families; 27.5% had skin lesions, 13.3% were positive for *O. ophiodiicola* DNA, and 77.8% of the qPCR positive individuals had skin lesions. This is the first report of *O. ophiodiicola* in five of the 22 species that were qPCR positive. Multinomial logistic regression modeling indicated that *Drymarchon couperi* had a higher relative risk of apparent ophidiomycosis (lesions present and qPCR positive), and the best models predicting qPCR result and ophidiomycosis category included individual factors and excluded temporal and spatial factors. Phylogeny-based bipartite network analysis showed that *Nerodia erythrogaster*, *Nerodia taxispilota*, and *D. couperi* had the highest prevalence of apparent ophidiomycosis; this category was more prevalent in the subfamily Colubrinae and less prevalent in Natricinae. These results provide important information about ophidiomycosis epidemiology, which has implications for snake conservation.

## Introduction

Fungal diseases have become increasingly prevalent in wildlife species over the past two decades^1^. Chytridiomycosis, caused by the fungus *Batrachochytrium dendrobatidis* (*Bd*), has been implicated as the cause of severe population declines in frogs worldwide^2^, and the related pathogen *Batrachochytrium salamandrivorans* has led to local extinctions of newts and salamanders in Europe^3^. Similarly, white nose syndrome in bats, caused by *Pseudogymnoascus destructans*, has emerged in the United States since 2006, spreading to 19 US states and killing over 5 million insectivorous bats^4^. In combination with factors such as habitat destruction and climate change, these fungal diseases pose extremely significant threats to biodiversity and ecosystem health.

Ophidiomycosis (also known as snake fungal disease; SFD) is an infectious disease of wild and captive snakes that threatens snake health worldwide^5^. Experimental infection studies have determined that the causative agent is the keratinophilic fungus *Ophidiomyces ophiodiicola*^6,7^, and the disease has been documented in more than 30 species of wild snakes in the United States and Europe^8–10^. Historically, *O. ophiodiicola* was first isolated from captive snakes with skin lesions in the United Kingdom and the United States in the mid-1980s^11^. Fungal isolates were obtained from captive snakes in Europe and Australia prior to the emergence of ophidiomycosis in wild snakes from North America, which is an important consideration in the epidemiology of the disease^11,12^. In the United States, ophidiomycosis has been identified in species of conservation concern, including timber rattlesnakes (*Crotalus horridus*)^13^, eastern massasauga rattlesnakes (*Sistrurus catenatus*)^14^, and eastern indigo snakes (*Drymarchon couperi*)^15^. Clinical signs of ophidiomycosis can vary significantly between individuals, from general signs such as lethargy, accelerated shedding cycles, and dysecdysis, to displaced or discolored scales, crusts, granulomas, and ulcers on the head and body. While lesions are typically confined to the skin, the fungus can invade into deeper tissues and granulomas have been found in internal organs such as the lungs, liver, and kidneys^5^. Infection can be fatal when granulomas result in organ failure or when lesions interfere with the animal’s ability to secure prey, evade predators, or protect itself from severe weather conditions^10^. Snakes can be assigned to ophidiomycosis categories based on the presence of skin lesions and detection of *O. ophiodiicola*^5^.

To date, most published research examining ophidiomycosis has centered on a single species of conservation concern^16^, focused on positive cases^10^, or examined relatively small groups of species^17^. While informative, these studies do not provide data regarding the overall epidemiology of the disease, including the impacts of geographic, temporal, and phylogenetic factors. Such broad examinations of disease distribution are crucial for informing management decisions that may mitigate the effects of ophidiomycosis on species of conservation concern and all snake populations worldwide^18^. Previous published reports of the disease in wild-caught snakes in the state of Georgia are rare, but include a black rat snake (*Pantherophis obsoletus*) with swelling around the right eye^19^, a mud snake (*Farancia abacura*) with head swelling and dysecdysis^20^, and populations of eastern indigo snakes (*Drymarchon couperi*), which are a species of conservation concern^15^. While these case reports indicate that the disease is present in wild snakes in the state, they do not provide information about the overall distribution and prevalence of the disease in wild populations. The purpose of this study was to investigate the species of free-ranging snakes impacted by ophidiomycosis in southeast Georgia, USA. We hypothesized that the prevalence of skin lesions, *O. ophiodiicola* DNA, and ophidiomycosis categories would be associated with temporal, spatial, and individual factors, including month, year, county sampled, sex, age class, and snake taxonomic group.

## Results

**Figure 1:**
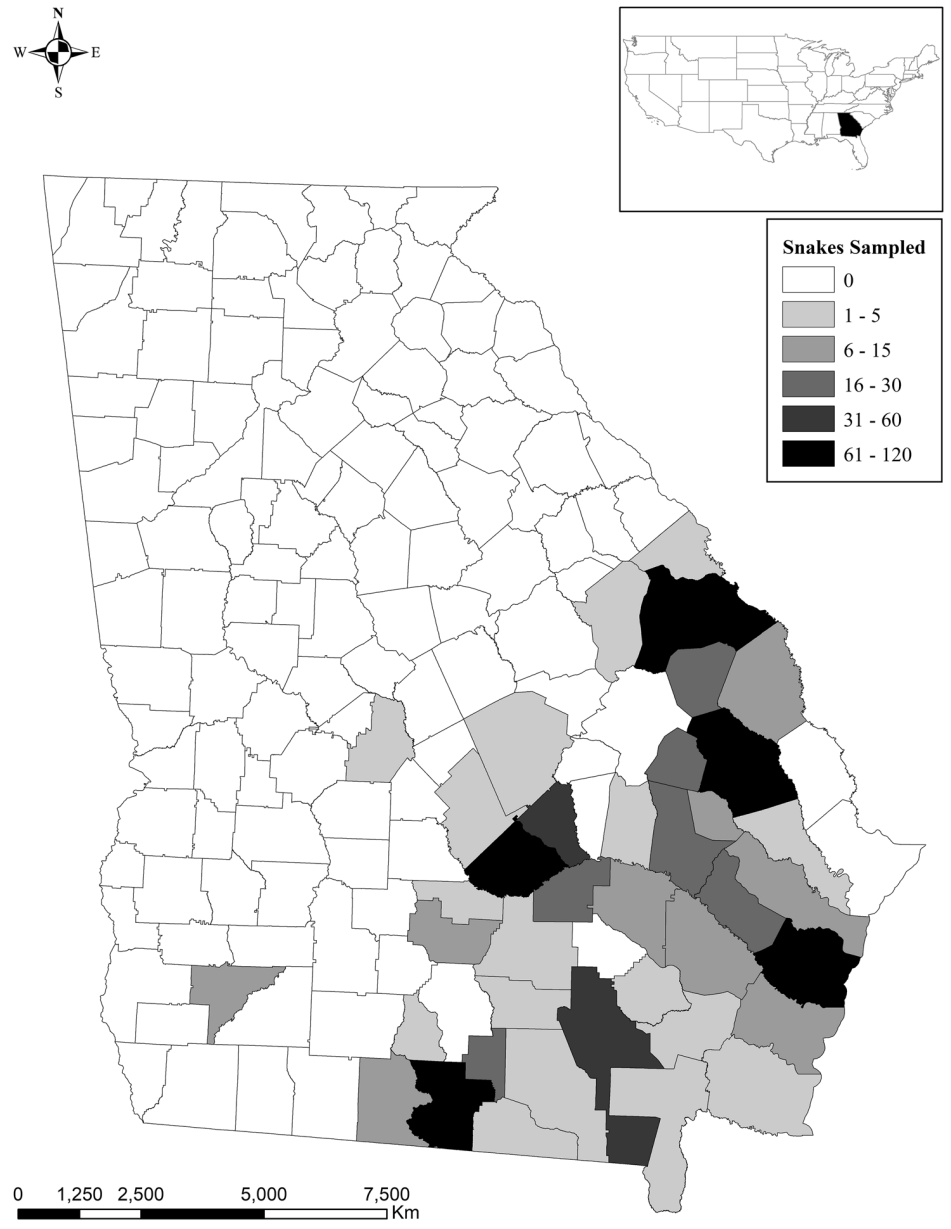
Map of counties in southeast Georgia, USA indicating number of snakes per county sampled for ophidiomycosis from 2016 to 2018. All species are included.

**Figure 2:**
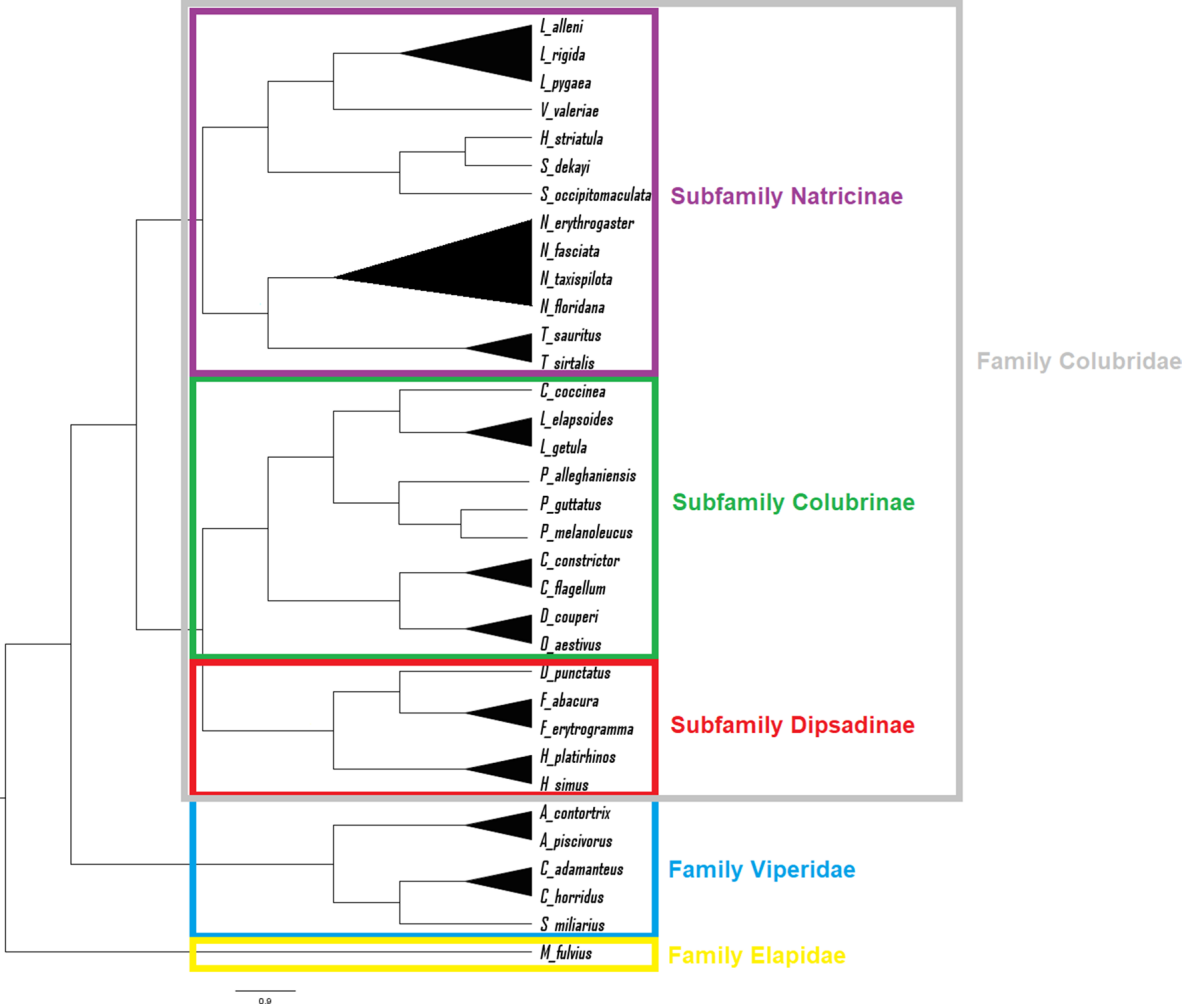
Phylogenetic reconstruction using the maximum likelihood algorithm and all partial cDNA sequences. Triangles represent species of the same genus grouped together. This tree shows relationships consistent with established taxonomy among 34 species, 3 families, and 3 subfamilies of Colubridae.

**Figure 3:**
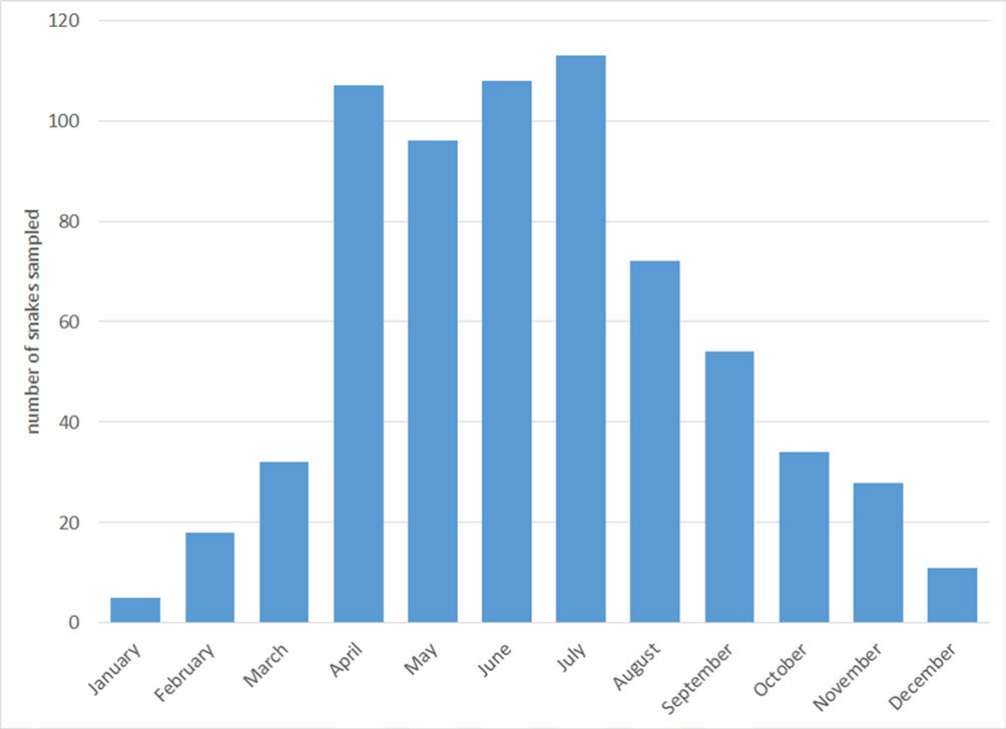
Bar graph of monthly sample sizes for snakes sampled for ophidiomycosis in Georgia, USA from 2016 to 2018. Eastern indigo snakes (*Drymarchon couperi*) are excluded.

A total of 962 swabs were collected from 786 individual snake encounters, including 107 eastern indigo snake encounters for which the results have been previously published^15^. Fifteen PIT-tagged eastern indigo snakes were sampled multiple times while recaptures of other species were unknown due to lack of permanent identification. Sampled snakes were from 39 counties (Fig. 1) and represented 34 species from 19 genera and 3 families (Table 1, Fig. 2). The majority of snakes were in the family Colubridae (n = 674), followed by Viperidae (n = 108), and Elapsidae (n = 4). Within the Colubridae, the subfamilies Colubrinae (n = 362), Natricinae (n = 232), and Dipsadinae (n = 80) were represented. Excluding eastern indigo snakes, most snakes were sampled in 2017 (n = 402), followed by 2018 (n = 254), and 2016 (n = 23). The months with the greatest number of snake captures were July (n = 113), June (n = 108), April (n = 107), and May (n = 96), followed by August (n = 72), September (n = 54), October (n = 34), March (n = 33), November (n = 28), February (n = 18), December (n = 11), and January (n = 5) (Fig. 3). There were 254 females, 189 males, and 236 snakes of unknown sex that included 428 adults, 150 juveniles, and 101 subadults.

**Table 1:** Sample size and prevalence of ophidiomycosis categories for snake species sampled in Georgia from 2016 to 2018. Species in bold do not have previously published reports of ophidiomycosis in wild snakes. Underlined species were excluded from the statistical analysis. Total weighted prevalence is shown for each category, including all species. 95% confidence intervals for prevalence are shown in parentheses.

Species	Scientific Name	Number of snakes sampled	Apparent ophidiomycosis prevalence (%)	Possible ophidiomycosis prevalence (%)	*Ophidiomyces* present prevalence (%)
Copperhead	*Agkistrodon contortrix*	13	7.7 (0.2–36)	7.7 (0.2–36)	0.0 (0–24.7)
Cottonmouth	*Agkistrodon piscivorus*	39	20.5 (9.3–36.5)	12.8 (4.3–27.4)	7.7 (1.6–20.9)
**Scarlet snake**	***Cemophora coccinea***	28	3.6 (0.1–18.3)	7.1 (0.9–23.5)	0.0 (0–12.3)
Eastern Racer	*Coluber constrictor*	57	12.3 (5.1–23.7)	17.5 (8.7–29.9)	0.0 (0–6.3)
Eastern Coachwhip	*Coluber flagellum*	25	0.0 (0–13.7)	40.0 (21.1–61.3)	0.0 (0–13.7)
Eastern Diamondback rattlesnake	*Crotalus adamanteus*	25	24.0 (9.4–45.1)	4.0 (0.1–20.4)	0.0 (0–13.7)
Timber rattlesnake	*Crotalus horridus*	15	0.0 (0–21.8)	6.7 (0.2–31.9)	13.3 (1.7–40.5)
Ring-necked snake	*Diadophis punctatus*	11	9.1 (0.2–41.3)	18.2 (2.3–51.8)	0.0 (0–28.5)
Eastern Indigo snake	*Drymarchon couperi*	107	43.0 (33.5–52.9)	40.2 (30.8–50.1)	0.0 (0–3.4)
Mud snake	*Farancia abacura*	27	14.8 (4.2–33.7)	29.6 (13.8–50.2)	0.0 (0–12.8)
**Rainbow snake**	***Farancia erytrogramma***	4	25.0 (0.6–80.6)	25.0 (0.6–80.6)	0.0 (0–60.2)
Rough earth snake	*Haldea striatula*	2	0.0 (0–84.2)	50.0 (1.3–98.7)	0.0 (0–84.2)
Eastern Hognose snake	*Heterodon platirhinos*	35	2.9 (0.1–14.9)	11.4 (3.2–26.7)	0.0 (0–10.0)
Southern Hognose snake	*Heterodon simus*	3	0.0 (0–70.8)	33.3 (0.8–90.6)	0.0 (0–70.80
Scarlet kingsnake	*Lampropeltis elapsoides*	8	0.0 (0–36.9)	0.0 (0–36.9)	0.0 (0–36.9)
**Eastern kingsnake**	***Lampropeltis getula***	16	12.5 (1.6–38.3)	12.5 (1.6–38.3)	18.8 (4.0–45.6)
Striped crayfish snake	*Liodytes alleni*	4	0.0 (0–60.2)	25.0 (0.6–80.6)	0.0 (0–60.2)
Black swamp snake	*Liodytes pygaea*	15	0.0 (0–21.8)	6.7 (0.2–31.9)	0.0 (0–21.8)
Glossy Crayfish snake	*Liodytes rigida*	20	0.0 (0–16.8)	25.0 (8.7–49.1)	0.0 (0–16.8)
Eastern coral snake	*Micrurus fulvius*	4	0.0 (0–60.2)	0.0 (0–60.2)	0.0 (0–60.2)
Plain-bellied watersnake	*Nerodia erythrogaster*	27	37.0 (19.4–57.6)	22.2 (8.6–42.3)	7.4 (0.9–24.3)
Banded watersnake	*Nerodia fasciata*	69	5.8 (1.6–14.2)	13.0 (6.1–23.3)	4.3 (0.9–12.2)
Florida Green watersnake	*Nerodia floridana*	2	0.0 (0–84.2)	50.0 (1.3–98.7)	0.0 (0–84.2)
Brown watersnake	*Nerodia taxispilota*	33	33.3 (18.0–51.8)	12.1 (3.4–28.2)	12.1 (3.4–28.2)
**Rough green snake**	***Opheodrys aestivus***	19	5.3 (0.1–26.0)	42.1 (20.3–66.5)	0.0 (0–17.6)
Eastern ratsnake	*Pantherophis alleghaniensis*	61	11.5 (4.7–22.2)	27.9 (17.1–40.8)	1.6 (0–8.8)
Corn snake	*Pantherophis guttatus*	32	0.0 (0–10.9)	9.4 (2.0–25.0)	3.1 (0.1–16.2)
**Pine snake**	***Pituophis melanoleucus***	9	11.1 (0.3–48.2)	33.3 (7.5–70.1)	0.0 (0–33.6)
Pygmy rattlesnake	*Sistrurus miliarius*	16	6.3 (0.2–30.2)	6.3 (0.2–30.2)	0.0 (0–20.6)
Brown snake	*Storeria dekayi*	2	0.0 (0–84.2)	0.0 (0–84.2)	0.0 (0–84.2)
Redbelly snake	*Storeria occipitomaculata*	2	0.0 (0–84.2)	0.0 (0–84.2)	0.0 (0–84.2)
Ribbon snake	*Thamnophis sauritus*	19	5.3 (0.1–26.0)	21.1 (6.1–45.6)	0.0 (0–17.6)
Garter snake	*Thamnophis sirtalis*	36	5.6 (0.7–18.7)	13.9 (4.7–29.5)	2.8 (0.1–14.5)
Smooth earth snake	*Virginia valeriae*	1	0.0 (0–97.5)	0.0 (0–97.5)	0.0 (0–97.5)
	**Overall**	**786**	**14.8 (12.3–17.4)**	**20.4 (17.5–23.3)**	**2.5 (1.6–3.9)**

Among species other than eastern indigo snakes (n = 679), the overall prevalence of skin lesions was 27.5% (n = 187; 95% CI 24.2–31.1%) and the prevalence of *O. ophiodiicola* DNA detection was 13.3% (n = 90; 95% CI 10.8–16.0%). Of the 90 qPCR positive individuals, 70 had skin lesions (77.8%, 95% CI 67.8–85.9%). *Ophidiomyces ophiodiicola* DNA was detected in 22 of the sampled species, including five species for which this is the first published report of ophidiomycosis in free-ranging individuals (Table 1). Sampled snakes were assigned to each of the four ophidiomycosis categories: most animals were negative (n = 472), followed by possible ophidiomycosis (n = 117), apparent ophidiomycosis (n = 70), and *Ophidiomyces* present (n = 20). Interestingly, there was a high prevalence of possible ophidiomycosis in *Opheodrys aestivus* (42.1%), *Drymarchon couperi* (40.2%), *Coluber flagellum* (40.0%), *Pituophis melanoleucus* (33%), and *Farancia abacura* (29.6%) (Table 1). There was no difference in standardized *O. ophiodiicola* copy number between species (*p* = 0.518) and the median standardized copy number for all positive swabs (n = 172) was 24.19 copies/ng DNA (10–90th percentiles: 0.91–595.18 copies/ng, range = 0.025–22,612.89 copies/ng). DNA concentration distributions were similar between positive (median = 5.81 ng/µl, 10th–90th percentiles: 2.05–9.76 ng/µl, range = 0.81–69.88 ng/µl) and negative samples (median = 3.29 ng/µl, 10th–90th percentiles: 1.66–6.8 ng/µl, range = 0.73–72.87 ng/µl), thus, negative results are unlikely to be a result of low DNA quantity.

### Generalized linear regression modeling

The top multivariable model for skin lesion presence was highly supported (Akaike weight = 0.999) and included the additive effects of year, month, species, and age class (Table 2). Significant predictors from the most parsimonious model recalculated using the maximum available data (n = 640 snakes) included year (variable DF = 2, model DF = 602, *p* = 0.0008), month (variable DF = 10, model DF = 602, *p* = 0.0001), age class (variable DF = 2, model DF = 602, *p* < 0.0001), and species (variable DF = 23, model DF = 602, *p* < 0.0001). The odds of lesions being present in 2018 were 2.51 times higher than in 2017 (95% CI 1.71–4.77, *p* = 0.0004) (Supplementary Fig. S1a). The odds of lesion presence were higher in March compared to April (OR 5.64, 95% CI 1.15–27.61, *p* = 0.02), May (OR 5.47, 95% CI 1.07–27.85, *p* = 0.03), June (OR 6.93, 95% CI 1.37–35.19, *p* = 0.006), July (OR 9.17, 95% CI 1.76–47.72, *p* = 0.0008), August (OR 5.91, 95% CI 1.04–33.51, *p* = 0.04), and September (OR 7.81, 95% CI 1.06–57.42, *p* = 0.04) (Supplementary Fig. S1b). The odds of lesion presence were 3.44 times higher in adults than juveniles (95% CI 1.74–6.81, *p* = 0.0001) (Supplementary Fig. S1c) and 15.34 times higher in *N. erythrogaster* than *P. guttatus* (95% CI 1.07–219.21, *p* = 0.04) (Supplementary Fig. S1d).

**Table 2:** AIC table of generalized linear models predicting (a) the presence of skin lesions, (b) qPCR detection of *Ophidiomyces ophiodiicola*, and (c) ophidiomycosis category in snakes from Georgia, USA, 2016–2018 (n = 618). Y = year, M = month, S = species, G = Genus, A = age class, K = number of parameters, AIC_c_ = second-order Akaike information criterion, ΔAIC_c_ = difference in AIC_c_ between ranked models, w_i_ = Akaike weight.

Model	Hypothesis	K	AIC_c_	ΔAIC_c_	w_i_
**(a) Skin Lesion Presence**					
Y + M + S + A	Temporal + Individual	38	680.3	0	0.999
Y + M + G + A	Temporal + Individual	29	694.5	14.26	0.001
S + A	Individual	26	699.7	19.41	0
Y + M	Temporal	13	708.6	28.38	0
G + A	Individual	17	716.5	36.28	0
Null	–	1	731	50.75	0
**(b) qPCR Detection of** *Ophidiomyces ophiodiicola*					
S + A	Individual	26	457.5	0	0.918
M + S + A	Temporal + Individual	36	463.5	6.03	0.045
G + A	Individual	17	463.9	6.45	0.036
M + G + A	Temporal + Individual	27	472.1	14.68	0.001
M + G + A + C	Temporal + Spatial + Individual	45	483.8	26.36	0
M + S + A + C	Temporal + Spatial + Individual	54	486.9	29.44	0
Null	–	1	500.7	43.18	0
M	Temporal	11	501.7	44.23	0
C	Spatial	19	503	45.56	0
**(c) Ophidiomycosis Category**					
S + A	Individual	78	1112.9	0	0.531
G + A	Individual	51	1113.2	0.3	0.458
Null	–	3	1121.2	8.27	0.008
Y + M	Temporal	39	1124.3	11.31	0.002
Y + M + G + A	Temporal + Individual	87	1135.1	22.16	0
Y + M + S + A	Temporal + Individual	114	1143.1	30.16	0
C	Spatial	57	1158.8	45.82	0
Y + M + G + A + C	Temporal + Spatial + Individual	141	1226.6	113.63	0
Y + M + S + A + C	Temporal + Spatial + Individual	168	1269.2	156.3	0

The top multivariable model for qPCR status was highly supported (Akaike weight = 0.918) and included the additive effects of species and age class (Table 2). Significant predictors from the most parsimonious model recalculated using the maximum available data (n = 640 snakes) included age class (variable DF = 2, model DF = 602, *p* = 0.0002) and species (variable DF = 23, model DF = 602, *p* < 0.0001). The odds of testing qPCR positive were 5.61 times higher in adults than juveniles (95% CI 2.06–15.31, *p* = 0.0002) and 3.78 times higher in subadults than juveniles (95% CI 1.11–12.86, *p* = 0.03) (Supplementary Fig. S2a). While species was a biologically important predictor of qPCR status, no between-species contrasts were statistically significant (Supplementary Fig. S2b).

The top two multinomial logistic regression models predicting ophidiomycosis category, excluding eastern indigo snakes, were both highly supported: the model containing the additive effect of species and age class carried slightly more weight (Akaike weight = 0.531) than the model containing the additive effects of genus and age class (Akaike weight = 0.458) (Table 2). Significant predictors from the most parsimonious model recalculated using the maximum available data (n = 640 snakes) included age class (variable DF = 6, model DF = 527, *p* < 0.0001) and species (variable DF = 69, model DF = 527, *p* < 0.0001). The relative risk of apparent ophidiomycosis compared to negative classification was significantly higher in adult snakes compared to juveniles (RR 7.03, 95% CI 3.86–12.94, *p* = 0.0001). It was also significantly higher in *N. erythrogaster* compared to *H. platirhinos* (RR 34.02, 95% CI 3.68–436.25, *p* = 0.002), *N. fasciata* (RR 13.61, 95% CI 3.31–142.37, *p* = 0.0003)*, T. sirtalis* (RR 18.56, 95% CI 3.24–186.1, *p* = 0.001), *C. coccinea* (RR 35.22, 95% CI 3.78–478.09, *p* = 0.002), and *C. constrictor* (RR 7.89, 95% CI 2.25–38.68, *p* = 0.001) (Supplementary Fig. S3).

In order to obtain a deeper understanding of ophidiomycosis differences between species, the most parsimonious model for predicting ophidiomycosis category was repeated including data from eastern indigo snakes. Statistically significant predictors in this model (n = 762) included age class (variable DF = 6, model DF = 681, *p* < 0.0001) and species (variable DF = 72, model DF = 681, *p* < 0.0001). The relative risks of apparent ophidiomycosis (RR 7.04, 95% CI 2.81–53.55, *p* < 0.0001) and possible ophidiomycosis (RR 2.52, 95% CI 1.41–4.94, *p* = 0.002) compared to ophidiomycosis negative classification were significantly higher in adult snakes than in juveniles. The relative risks of possible and apparent ophidiomycosis, compared to negative, were higher in *D. couperi* than in most other species (Table 3, Supplementary Fig. S4). The relative risk of apparent ophidiomycosis compared to negative classification was also higher in *N. erythrogaster* than *H. platirhinos*, *N. fasciata, T. sirtalis*, *C. coccinea*, and *C. constrictor* and higher in *N. taxispilota* than *N. fasciata* (Table 3, Supplementary Fig. S4). Contingency tables for significant predictors in each of the models are included in the Supplementary Information (Supplementary Tables S1–S14).

**Table 3:** Risk ratios for ophidiomycosis category by species. NS = non-significant, dashes indicate absence of analysis due to structural zeros.

Species	vs.	Possible ophidiomycosis vs. negative			Apparent ophidiomycosis vs. negative		
		Risk ratio	95% Confidence interval	*p* value	Risk ratio	95% Confidence interval	*p* value
*D. couperi*	*A. piscivorus*	8.8	(2.9–27.2)	0.0001	5.4	(2–14.7)	0.001
*D. couperi*	*C. adamanteus*	47	(5.8–380.2)	< 0.0001	9.3	(3.2–27.5)	< 0.0001
*D. couperi*	*C. coccinea*	27	(5.7–127)	< 0.0001	57.7	(7.2–463)	< 0.0001
*D. couperi*	*C. constrictor*	8.4	(3.5–20.7)	< 0.0001	12.8	(4.8–34.4)	< 0.0001
*D. couperi*	*H. platirhinos*	14.2	(4.3–46.9)	< 0.0001	56.1	(7–449.5)	< 0.0001
*D. couperi*	*L. pygaea*	32.2	(3.9–264.7)	0.001	–	–	–
*D. couperi*	*N. fasciata*	10.7	(4.3–26.7)	< 0.0001	22.9	(7.1–74.2)	< 0.0001
*D. couperi*	*P. alleghaniensis*	4.3	(1.9–9.7)	< 0.0001	10.6	(3.9–28.8)	< 0.0001
*D. couperi*	*P. guttatus*	20.6	(5.5–76.8)	< 0.0001	–	–	–
*D. couperi*	*S. miliarius*	33.8	(4.1–277.7)	0.001	38.5	(4.7–317.6)	0.0006
*D. couperi*	*T. saurita*	6.9	(2–24.3)	0.002	27.7	(3.3–231.1)	0.002
*D. couperi*	*T. sirtalis*	11.6	(3.8–35.3)	< 0.0001	30.1	(6.4–142)	< 0.0001
*N. erythrogaster*	*H. platirhinos*	NS	NS	NS	33.7	(3.6–312)	0.002
*N. erythrogaster*	*N. fasciata*	NS	NS	NS	13.8	(3.4–56.6)	0.0003
*N. erythrogaster*	*T. sirtalis*	NS	NS	NS	18.1	(3.2–103.4)	0.001
*N. erythrogaster*	*C. coccinea*	NS	NS	NS	34.7	(3.7–323)	0.002
*N. erythrogaster*	*C. constrictor*	NS	NS	NS	7.7	(2.2–27.3)	0.002
*N. taxispilota*	*N. fasciata*	NS	NS	NS	22.9	(26.4–82.2)	0.0007

### Phylogeny reconstruction

Six different consensus phylogenetic trees were obtained: three based on partial protein sequences (Supplementary Fig. S5) and three based on partial nucleotide sequences (Supplemental Fig. S6). The trees were evaluated based on how frequently snake species belonging to the same genus were grouped together, as indicated by triangles on each tree. The maximum likelihood tree using partial nucleotide sequence data grouped species within the same genus, subfamily and family according to established snake taxonomy^21^ (Fig. 2), so it was used as the framework for building the network.

### Network analysis

**Figure 4:**
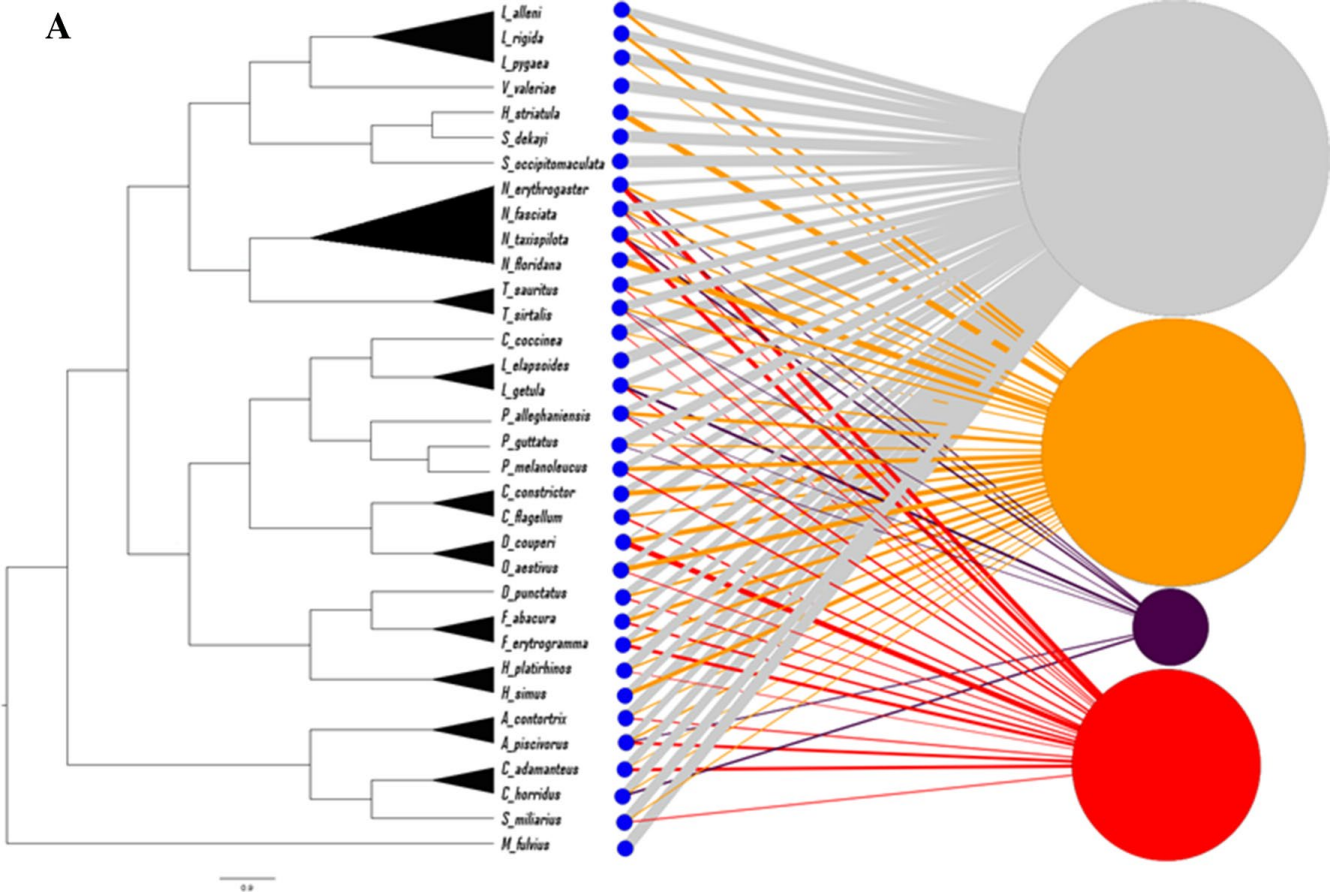

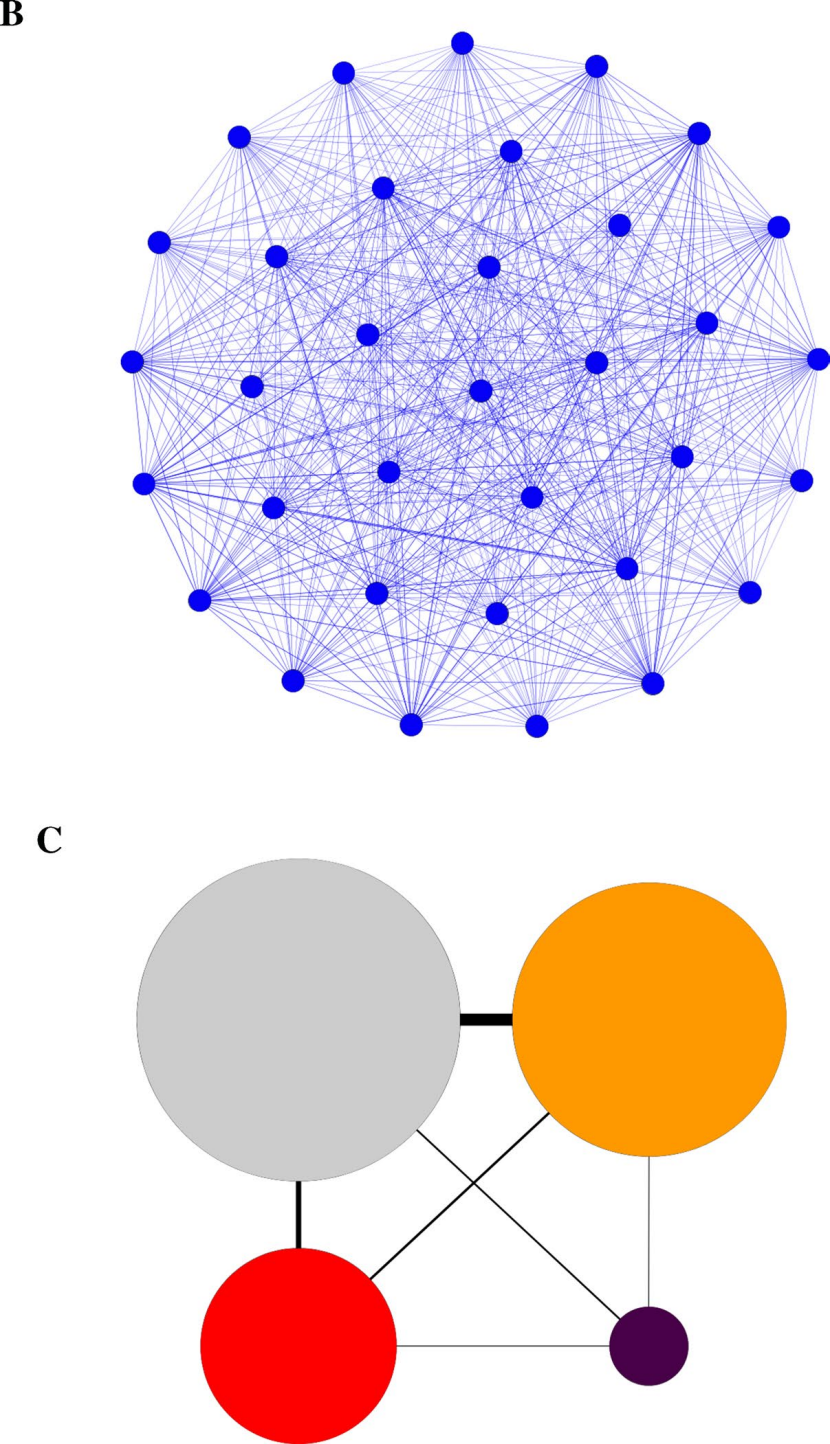
Bipartite network and network projections of snakes sampled for ophidiomycosis in Georgia 2016–2018. The network is built based on proportion of snakes of each species in each of four ophidiomycosis categories, where blue nodes represent sampled snake species, the gray node represents the negative category, the orange node represents possible ophidiomycosis, the dark purple node represents *Ophidiomyces* present, and the red node represents apparent ophidiomycosis. Node size is weighted by prevalence of the ophidiomycosis category. (**a**) Bipartite network with species nodes aligned with the corresponding leaves of the best phylogenetic tree. Nodes are connected if snakes of a given species were classified into the given category, and links are weighted by the proportion of snakes of the given species in the given category. (**b**) Species-species network projection with species nodes connected if they share a connection with an ophidiomycosis category, (**c**) Disease-disease network projection with ophidiomycosis category nodes connected if they share a link with a snake species.

**Figure 5:**
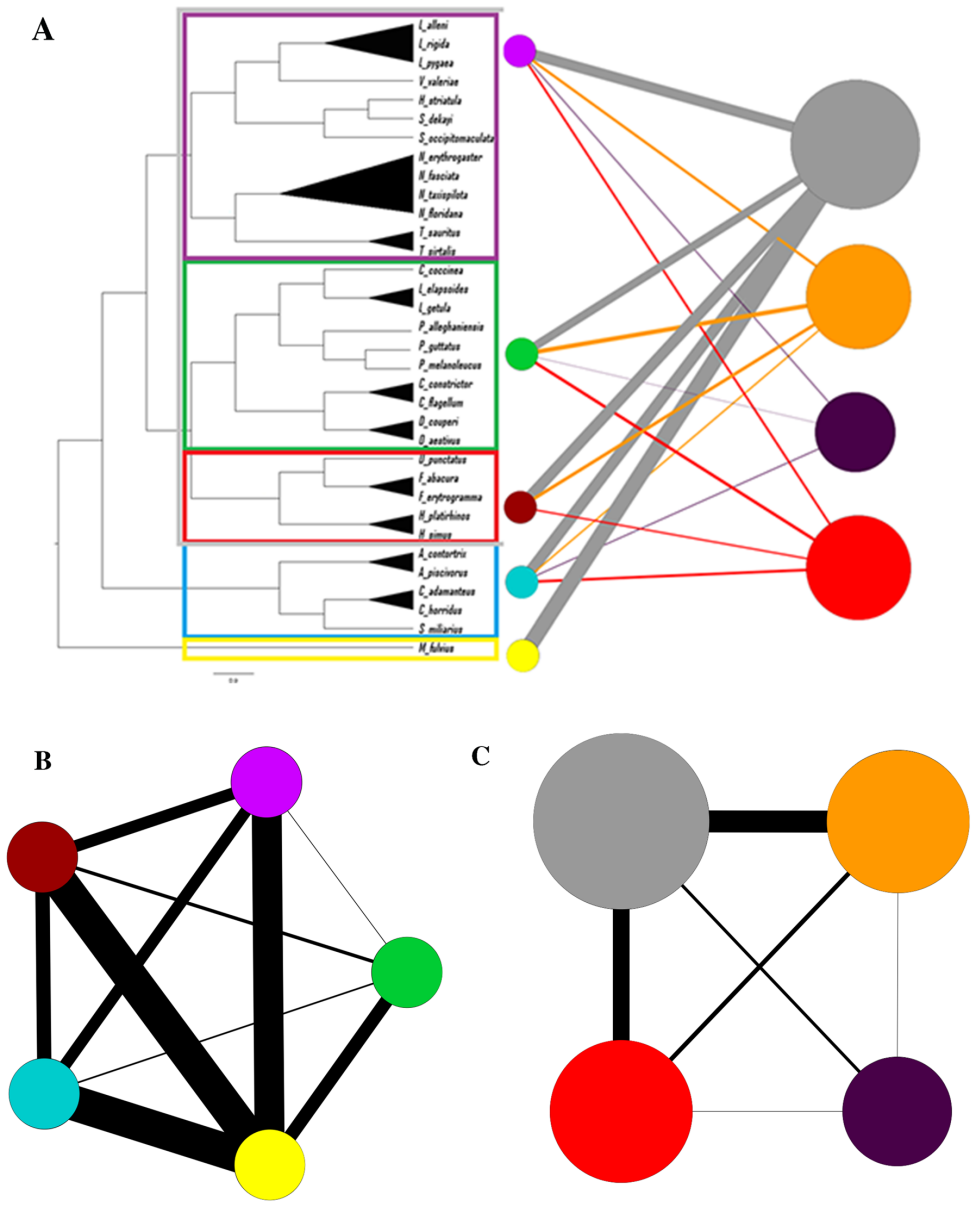
Bipartite network and projections with snake species grouped by family/subfamily for snakes sampled for ophidiomycosis in Georgia 2016–2018. The purple, green, and maroon nodes represent the subfamilies Natricinae, Colubrinae, and Dipsadinae of the family Colubridae, the turquoise node represents the family Viperidae, and the yellow node represents the family Elapidae. The gray node represents the ophidiomycosis negative category, the orange node represents possible ophidiomycosis, the dark purple node represents *Ophidiomyces* present, and the red node represents apparent ophidiomycosis. Ophidiomycosis category node size is proportional to the prevalence of the category. (**a**) Bipartite network aligned with the best phylogenetic tree showing each family/subfamily, nodes are connected if snakes of a given family/subfamily were classified into the given ophidiomycosis category, and the links are weighted based on the proportion of snakes of the given family/subfamily in the given category, (**b**) Family-family network projection with species nodes connected if they share a connection with an ophidiomycosis category, (**c**) Disease-disease network projection with disease nodes connected if they share a link with a snake family/subfamily.

Two bipartite networks were created, each with two projections. The first network (Fig. 4a) shows the proportion of snakes in each species that were classified into each ophidiomycosis category, with the species nodes aligned to the leaves of the phylogenetic tree. A high proportion of all species were classified as negative, thus the largest ophidiomycosis node was the negative category, followed by possible ophidiomycosis, then apparent ophidiomycosis, and finally *Ophidiomyces* present. The strongest connections to the apparent ophidiomycosis group are from *N. erythrogaster, N. taxispilota,* and *D. couperi*, while numerous species have no connection to apparent ophidiomycosis category, including the genera *Liodytes, Haldea,* and *Storeria*. Both the species-species projection (Fig. 4b) and the disease-disease projection (Fig. 4c) show high connectivity between nodes, and the disease-disease projection indicates the strongest connection between the negative, possible ophidiomycosis, and apparent ophidiomycosis categories. The second network based on family/subfamily (Fig. 5a) shows connections between every family/subfamily and every ophidiomycosis category, except that the Elapsidae family only had snakes in the negative category, and the Dipsadinae family did not have any snakes in the *Ophidiomyces* present category. Again, both the family-family projection (Fig. 5b) and the disease-disease projection (Fig. 5c) showed high connectivity, and the family-family projection shows the weakest link between the Natricinae and Colubrinae subfamilies of Colubridae. In most cases, snakes of the same species/subfamily/family were classified into multiple ophidiomycosis categories, which resulted in high interconnectedness among nodes in the network projections.

## Discussion

In this study, *O. ophiodiicola* DNA was detected in 22 snake species in Georgia, USA, five of which have not been previously reported with the pathogen in the wild. Of these species, eastern kingsnakes (*Lampropeltis getula*), rough green snakes (*Opheodrys aestivus*), and pine snakes (*Pituophis melanoleucus*) have been previously tested for ophidiomycosis in our lab and have either been wild snakes with negative results or captive snakes with positive results (M.C. Allender, unpublished data). To our knowledge, the remaining species have not been previously tested. Our statistical modeling indicated that *D. couperi* had a higher relative risk of possible ophidiomycosis, and, along with *N. erythrogaster* and *N. taxispilota*, had a higher relative risk of being in the apparent ophidiomycosis category, compared to the negative category. The phylogeny-based bipartite network analysis supported these findings, showing the strongest connections between apparent ophidiomycosis and the aforementioned three species. In general, there were more connections to apparent ophidiomycosis from species in the subfamily Colubrinae and fewer from species in the subfamily Natricinae, including the genera *Liodytes*, *Haldea*, and *Storeria*. *Nerodia* species have previously been shown to have a high prevalence of ophidiomycosis and appear uniquely sensitive to infection^10,22^. However, the relative risk of apparent ophidiomycosis was higher in *N. erythrogaster* and *N. taxispilota* compared to *N. fasciata*, indicating that there may be differences in susceptibility even within genera. The lower observed prevalence in certain species and genera may be due to inherent resistance shared among closely related species, the smaller size of the snake resulting in smaller surface area for sampling, or sharing life history traits/habitats that are less permissive to developing ophidiomycosis. One potential explanation for differences in disease prevalence involves differences in the structure of the skin, as skin is an important immune barrier to microbial invasion^23^ and skin thickness has been found to vary among snakes with different life histories^24^. Future investigations are needed to characterize the mechanisms leading to disease prevalence differences between species and should continue to sample these species in the wild, evaluate environmental factors that influence snakes’ exposure to the fungus, and conduct challenge studies to evaluate disease progression and host immune responses.

It is of particular note that the top model for lesion presence included temporal and individual factors, while the top models for qPCR result and ophidiomycosis category only included individual factors. None of the top models included spatial factors. These results support the hypothesis that ophidiomycosis predisposition is based more on factors such as species and age class, rather than seasonality or geography. In comparison, ophidiomycosis category was found to associated with month, but not sex or age class, in eastern indigo snakes^15^. This may indicate that temporal factors are important on an individual species basis, since different species have different activity seasons. Further work is needed to investigate such trends.

The species-species (Fig. 4b) and family-family (Fig. 5b) projections of the bipartite networks illustrate connections between taxonomic groups based on shared ophidiomycosis categories, and the disease-disease projections (Figs. 4c and 5c) illustrate connections between disease categories based on the number of species or families shared between categories. Since snakes of every species were negative and most species had snakes in multiple categories, the nodes are highly interconnected. This shows how much individual variation in disease status exists across populations, which may be due to differences in susceptibility or each individual’s disease progression status at the time of sampling. Since the fewest snakes were categorized as *Ophidiomyces* present, this is the least connected node in the disease-disease projection, while the other nodes are more highly connected.

A recent analysis by Burbrink et al.^25^ evaluated 23 previously reported species of susceptible wild snakes in the eastern United States, with susceptibility defined as having characteristic fungal dermatitis and *O. ophiodiicola* cultured from the lesions, and found that ophidiomycosis susceptibility was not associated with phylogeny or ecological traits. In comparison to the study presented here, that analysis was limited to species with snakes in the apparent ophidiomycosis category and did not examine relative prevalence across species. Including additional species and ophidiomycosis categories in our analysis helps to illuminate more subtle trends, such as ophidiomycosis not affecting species in an all-or-nothing manner but rather on a scale of relative susceptibility. Susceptibility is likely multifactorial and may be related to phylogenetic and ecological factors, so additional studies are recommended to further investigate the epidemiology of the disease as described above.

The overall prevalence of skin lesions in this study, excluding eastern indigo snakes, was 27.5% and the prevalence of animals testing positive for *O. ophiodiicola* DNA was 13.3%. In eastern indigo snakes alone, 83.2% of snakes had skin lesions and 43.9% of snakes tested qPCR positive^15^. Previous state-wide studies found a 12.7% prevalence of skin lesions in eastern massasauga rattlesnakes in Michigan^16^, a 51.7% prevalence of lesions in snakes in Kentucky^18^, and a 30.0% prevalence of qPCR positive snakes in Tennessee^22^. However, the prevalence of disease in eastern massasaugas in Michigan was found to vary significantly across time and space, with the prevalence of lesions varying from 0 to 61.1% and the prevalence of positive qPCR results varying from 0 to 13.3%^26^. Disease prevalence estimates from single time points are of limited value for understanding ophidiomycosis as they are biased by seasonality of sampling, species sampled, progression through multiple stages of infection over time, and discrepancies between diagnostic methodologies. Based on our finding that certain species have a higher prevalence of disease, sampling efforts that include a larger proportion of predisposed species, such as *D*. *couperi*, would inherently have a higher prevalence than those that sample fewer of these species. Disease progression also makes it difficult to estimate the true prevalence of the disease when snakes are sampled at a single time point. In experimentally challenged cottonmouths (*Agkistrodon piscivorus*), clinical signs did not present until 1–2 months after challenge with *O. ophiodiicola*, and clinical signs in most snakes resolved prior to death or euthanasia^6^. This indicates that some snakes who appear healthy, but have DNA present on their skin, may be newly exposed and develop clinical signs later, or may have recovered from previous infection. Long-term monitoring of marked individuals, as has been done with eastern indigo snakes^15^, presents an ideal opportunity to examine how ophidiomycosis progresses in wild snakes. Dividing snakes into ophidiomycosis categories helps to clarify each animal’s disease status and, combined with mark-recapture and tracking studies, can help us to understand how wild snakes are impacted by the disease over time. While the frequency of recaptures in this study is known for eastern indigo snakes^15^, it is possible that snakes of other species were captured and sampled multiple times. We recognize this as a limitation of the study, since numerous recaptures of the same animals would bias prevalence estimates and the results of our analyses. However, we did not collect additional samples from snakes who were visually recognized as previously sampled and, based on a review of the data for mass, length, SVL, sex, and site for individual snakes, there are few snakes who may have been sampled multiple times. Therefore, it is unlikely that any recaptures had a statistically significant impact on the conclusions of this study and we recommend that future studies permanently identify all snakes in areas where multiple surveys will be conducted.

An additional limitation in applying the results of this study is the unequal sample sizes across species, geographic areas, and temporal scales. We were able to sample large numbers of certain species due to targeted surveillance, but only a few individuals of other species, due to factors such as cryptic life histories. Furthermore, snake taxon diversity is not equally distributed across the landscape and snakes are not equally active at all times of the year. While this limits the conclusions that can be drawn from this data set, the trends observed in this study are ecologically relevant and provide guidance for future work investigating the epidemiology of ophidiomycosis.

Diagnostic testing has been one of the most difficult aspects in characterizing the epidemiology of ophidiomycosis. It has been previously found that the rate of false negatives with swabbing is nearly 10 times higher in animals without lesions than individuals with lesions^26^. The current recommendation to reduce this false negative rate is to repeatedly and firmly swab along the entire skin surface of each snake. While sampling a single site with a single swab has been found to increase the probability of a false negative qPCR result compared to swabbing the snake’s entire body with multiple swabs^26^, recent work examining the microbiome of eastern massasauga rattlesnakes with ophidiomycosis found that *O. ophiodiicola* was detectable on body sites distant to lesions in affected animals^27^. Thus, a thorough swabbing of the head, as was done in this study with snakes lacking lesions, would be expected to yield a positive qPCR result if the animal has *O. ophiodiicola* on its body, even if the lesions were not on the head. An additional diagnostic challenge arises when animals with skin lesions have a negative qPCR result. This occurred with 159 animals in this study (20.2% of snakes sampled). Possible explanations for this include: (1) the difficulty in detecting fungal DNA via swabbing when DNA quantity is low or fungus is present in tissues deeper than the epidermis; (2) the presence of qPCR inhibitors in samples; (3) the similar appearance of ophidiomycosis to non-infectious skin disease such as trauma; and (4) the presence of a different pathogen causing similar skin lesions. As part of our sampling protocol for animals with observed lesions, lesion swabs were collected in addition to the head swab, which would decrease the likelihood of false negatives in these animals. Overall, prevalence estimates for ophidiomycosis in wild snakes provide incomplete information and future efforts should focus on determining the overall distribution of the disease by species, geographic area, and time, as well as population-level impacts of the disease.

Networks are important tools for analyzing the structure, function, and dynamics of a wide variety of systems and are extremely useful for identifying epidemiologic trends. Contact networks are commonly used to analyze disease transmission between individuals in both human^28^ and wildlife populations^29^. While this approach is extremely challenging in wild snakes, whose movements and interactions are difficult to track, bipartite networks can be used to examine connections between two sets of data, such as species and disease traits, and illuminate overall trends. These networks are defined by having two sets of nodes and no two nodes within the same set adjacent^30^. A bipartite network analysis conducted to look for associations with chytridiomycosis in frogs observed that skin sloughing rate varied with phylogenetic group, but there was no evidence that sloughing was associated with *Bd*-driven population declines^31^. Bipartite networks have been used extensively to examine the factors that influence human disease. Goh et al. created a “diseaseome” connecting diseases with genetic origins and genes with known disease links to illuminate the complex associations between human diseases and genes^32^. Genome-wide association studies have also been used to construct networks linking markers of genetic variation and disease phenotypes^33^. Specific gene-disease networks have been created for autoimmune, neurologic, and cardiovascular diseases^34–36^, and exposure-disease networks have been created to analyze interactions between environmental factors, genes, and disease^37^. Such “network medicine” allows physicians and researchers to better understand the molecular pathophysiology of disease, predict new disease susceptibility genes, and identify new targets for specific treatment^38^. Our bipartite networks were created as mathematically-based visualizations of the data collected in this study and, as such, complement the use of generalized linear modeling in identifying taxonomic trends in ophidiomycosis prevalence. Such trends are critical to informing future investigations and management strategies that aim to conserve more susceptible snake species. The results of this study represent the first step toward identifying species predilections that will aid in identifying specific genes associated with disease susceptibility. The genetic basis may be through immune response, physical characteristics such as skin thickness, life history traits, or other factors. The next steps, including full sequencing of wild snake genomes and genomic analysis of *O. ophiodiicola* isolates, will allow us to better understand the epidemiology of this disease and design treatments and management programs to protect snake health.

Investigating the epidemiology of ophidiomycosis requires collaboration by biologists, veterinarians, and land managers. Previous studies have documented the distribution of the disease, but there is still more work to be done on this and numerous other conservation threats to snakes. Wildlife are known to be sentinels for the health of domestic animals and people, so studies of wildlife diseases are critical for the early detection and prevention of future disease outbreaks. Continuing to assess health and pathogen prevalence in snake populations enables us to better understand both new and ongoing threats to snakes and other wildlife species.

## Methods

### Field surveys

Snakes were captured using multiple sampling techniques, including visual encounter surveys, road cruising surveys, cover boards, and drift fence arrays. Both targeted and opportunistic surveys were conducted to sample as many species of the snake fauna of southern Georgia as possible across a broad geographic area and in a wide variety of habitat types. Samples were collected from a total of 39 counties in Georgia from September 1, 2016 through August 11, 2018 (Fig. 1). At the time of capture, all animals were assessed for clinical signs suggestive of ophidiomycosis, including displaced/thickened scales, crusts, pustules, ulcers, and necrotic scales^5^, and the presence or absence of clinical signs was recorded for each animal. For individuals without lesions, a single swab was collected from the snake’s head using a sterile cotton-tipped applicator. For individuals with skin lesions consistent with ophidiomycosis, swabs were collected directly from the affected area(s), in addition to the head swab. After collection, swabs were placed in 2 ml Eppendorf tubes and frozen at -20 °C until analysis. Head swabbing was chosen as the surveillance sampling method based on the first reports of ophidiomycosis in eastern massasauga rattlesnakes, in which lesions were limited to the heads of affected animals^14^. While protocols developed after the start of this study recommend whole body swabbing^26^, sample location has been shown to not be a significant predictor of *O. ophiodiicola* detection^27^.

Eastern indigo snakes were implanted with subcutaneous passive integrated transponder (PIT) tags for permanent identification. While other species were not permanently identified, animals who were visually recognized as having already been captured and sampled were not sampled multiple times. All snakes were released near their point of capture, generally within an hour of capture. Biosecurity was maintained while collecting samples, including wearing gloves, sanitizing hands between snakes, and cleaning equipment with an alcohol or bleach solution according to previously published recommendations^39^. All animal activities were approved and permitted by the Georgia Department of Natural Resources (Scientific Collecting Permits 29–WJH–16–21, 029, and 115579244) and the United States Fish and Wildlife Service (USFWS Permit TE28025A-2). Snake handling and sampling were conducted following ethical guidelines of the USFWS and the University of Illinois.

### Sample analysis

DNA extraction and quantitative PCR amplification (qPCR) were performed to detect *O. ophiodiicola* DNA in swab samples. The primers OphioITS-F and OphioITS-R and the probe Probe-FAM were used in the qPCR protocol as previously described^40^. DNA extraction followed the manufacturer’s recommendations (QIAamp DNA mini Kit, Qiagen Inc., Valencia, CA) with the addition of a one-hour incubation at 37 °C with 12.5U of lyticase (Sigma-Aldrich, St. Louis, MO), prior to the lysis step, to break down the fungal cell wall. Following DNA extraction, each sample was assessed for DNA quantity (measured in ng/µl) and quality (using the ratio of absorbance at 260 nm to 280 nm) using spectrophotometry (Nanodrop1000, ThermoFisher Scientific, Wilmington, DE). qPCR was performed in triplicate on a QuantStudio3 Real Time PCR system (Applied Biosystems, Foster City, CA). Samples were considered positive if replicates had a mean cycle threshold (C_t_) value lower than the lowest detected standard dilution on the same plate. Mean fungal quantities (copies per reaction) were standardized to the total quantity of DNA in the sample by dividing the mean copies/µl for each sample by the DNA concentration, as determined by spectrophotometry, yielding standardized fungal quantities in copies per ng DNA.

### Data management

Epidemiologic data from the eastern indigo snakes sampled during this study have been published separately^15^. All analyses presented in this manuscript exclude data from eastern indigo snakes, except when describing the counties and species sampled or in cases where between-species comparisons are of interest, specifically phylogenetic tree construction, network analysis, and one multinomial logistical regression model comparing ophidiomycosis categories between species. For the purposes of statistical analysis, species with five or fewer individuals represented, counties with five or fewer animals sampled, and months with five or fewer animals sampled were also excluded.

### Statistical analysis

Each snake was assigned to one of four ophidiomycosis categories based on the presence of gross skin lesions and *O. ophiodiicola*: (1) Negative: no lesions and qPCR negative; (2) *Ophidiomyces* present: no lesions and qPCR positive; (3) Possible ophidiomycosis: lesions present and qPCR negative; (4) Apparent ophidiomycosis: lesions present and qPCR positive^5^. Prevalence of lesions, qPCR positive results, and ophidiomycosis category were estimated, including calculating 95% binomial confidence intervals^41^. Weighted average prevalence was calculated for each ophidiomycosis category. Normality of standardized fungal quantities (copies per ng DNA) was assessed using the Shapiro–Wilk test, then means and 95% confidence intervals were calculated and compared using a Kruskal Wallis test. Unless otherwise specified, statistical significance was assessed at α = 0.05. All statistical testing was conducted using R v. 3.5.1^42^.

Bias-reduced generalized linear models (R package brglm2)^43^ were used to model lesion presence (present/absent) and qPCR status (positive/negative). Post-hoc tests were performed with the contrast function in the R package lsmeans^44^, using a Tukey adjustment for multiple statistical comparisons. Odds ratios were calculated for significant predictors. Multinomial logistic regression models (function multinom, R package nnet)^45^ were used to model ophidiomycosis category (negative, possible ophidiomycosis, *Ophidiomyces* present, apparent ophidiomycosis) with a Bonferroni p-value adjustment. Risk ratios were calculated for significant predictors. Effect size plots were generated using the lsmeans^44^ and effects^46^ packages in R.

Predictor variables (year, month, county, genus, species, age class, and sex) with a univariable p-value < 0.2 were included in multivariable models predicting lesion presence, qPCR status, and ophidiomycosis category. Collinearity was assessed using variance inflation factors (function vif in R package car)^47^, and variables with unacceptable variance inflation (VIF > 10) were not considered together within the same model. Candidate model sets were designed to test specific biological hypotheses about the impacts of temporal, spatial, and individual factors. Case-wise deletion was pursued to remove observations with missing data prior to information-theoretic model ranking (MuMIn package)^48^. Following model ranking, the most parsimonious model for each dependent variable of interest (lesion presence, qPCR status, and ophidiomycosis category) was reproduced using the maximum available dataset. All effect sizes and variable significance are reported from these final models.

To compare ophidiomycosis categories among all sampled species, an additional multinomial logistic regression model was performed including data from *D. couperi*. Species and age class were the independent variables (mirroring the most parsimonious model for ophidiomycosis category in the dataset without indigo snakes), and ophidiomycosis category was the dependent variable.

### Phylogenetic analysis

A phylogenetic tree was created to examine the relationships between all species sampled in this study, including eastern indigo snakes. Partial nucleotide sequences were downloaded from NCBI batch entrez using previously published accession numbers for the following genes: 12S, 16S, BDNF, CMOS, CYTB, ND2, ND4, NT3^49^. A concatenation of these genes was previously used by Figueroa *et al.*^49^ to generate snake phylogeny, so this technique was used to generate a tree for a subset of snake species. Sequences were aligned using Clustal Omega^50^ and the NEXUS output files were concatenated using SequenceMatrix^51^. All sequences and alignments were manually checked and all gaps were set as missing data. The concatenated file was executed in PAUP (Version 4.0, Sinauer Associates, Sunderland, MA) to construct phylogenetic trees using the following three methods: maximum likelihood, maximum parsimony, and least squares distance. PAUP settings were designated as three bootstrapping replicates, a heuristic search using 1000 trees, generating a consensus tree for each method, and rooting the trees with *M. fulvius* as an outgroup, since that was the only species sampled in the family Elapsidae. Trees were viewed and edited for publication using FigTree (Version 1.4.4, https://tree.bio.ed.ac.uk/software/figtree/).

### Network analysis

Bipartite networks and network projections were created using Gephi software (version 0.9.2)^52^. In the first network, one set of nodes represented the snake species and the second set of nodes represented the four disease categories, as described above. Nodes were linked if snakes of the given species met the criteria of the given disease category, and the link thickness was weighted based on the proportion of snakes of the given species in the given disease category, similar to previous networks created for human diseases and disease genes^32,53^. In the second network, species were grouped by family or subfamily and the network was created with link thickness based on the proportion of snakes in each family/subfamily meeting the criteria for each disease category. Network projections were created using the MultiMode Projections window in Gephi. In the species-species network projections, species nodes were linked if they shared a disease category, and the weight of the connection was proportional to the number of shared categories. In the disease-disease projections, nodes were linked if they were connected to one or more of the same species, with the weight of the link proportional to the number of shared species.

## Supplementary information

Supplementary file1

## Data Availability

The data generated and analyzed during the current study are available from the corresponding author on reasonable request.
