# Publisher Correction: Ophidiomycosis surveillance of snakes in Georgia, USA reveals new host species and taxonomic associations with disease

**DOI:** 10.1038/s41598-020-69878-z

**Published:** 2020-09-16

**Authors:** Eellen Haynes, Houston C. Chandler, Benjamin S. Stegenga, Laura Adamovicz, Eemilie Ospina, Dessireé Zerpa‑Catanho, Dirk J. Stevenson, Matthew C. Allender

**Affiliations:** 1 Wildlife Epidemiology Laboratory, College of Veterinary Medicine, University of Illinois at Urbana-Champaign, Urbana, IL USA; 2 The Orianne Society, Tiger, GA USA; 3 Department of Plant Biology, University of Illinois Urbana-Champaign, Urbana, IL USA; 4 Altamaha Environmental Consulting, Hinesville, GA USA

Correction to: *Scientific Reports* 10.1038/s41598-020-67800-1, published online 02 July 2020

In the original version of this Article, Figure 4C was a duplication of Figure 1. This has now been corrected in both the HTML and PDF versions of the Article.

