# Supplementary material for: Ophidiomycosis surveillance of snakes in Georgia, USA reveals new host species and taxonomic associations with disease
Source: Sci Rep. 2020 Jul 2;10:10870. doi: 10.1038/s41598-020-67800-1 (PMC7331741; doi:10.1038/s41598-020-67800-1)
Supplement: Supplementary file 1 — Supplementary file1 [file 41598_2020_67800_MOESM1_ESM.pdf]

Ophidiomycosis surveillance of snakes in Georgia, USA reveals new host species and taxonomic associations with disease

Ellen Haynes*[1], Houston C. Chandler[2], Benjamin S. Stegenga[2], Laura Adamovicz[1], Emilie Ospina[1], Dessireé Zerpa-Catanho[3], Dirk J. Stevenson[2,4], Matthew C. Allender[1]

[1] Wildlife Epidemiology Laboratory, University of Illinois at Urbana-Champaign, College of Veterinary Medicine, Urbana, Illinois, USA

[2] The Orianne Society, Tiger, Georgia, USA

[3] Department of Plant Biology, University of Illinois Urbana-Champaign, Urbana, Illinois, USA

[4] Altamaha Environmental Consulting, Hinesville, Georgia, USA

***Corresponding author**: haynes11@illinois.edu

Supplementary Table S1: Contingency table showing number of snakes sampled for ophidiomycosis by species and age class in Georgia, USA from 2016 to 2018.

**Species**	**Number sampled**	**Juvenile**	**Subadult**	**Adult**
Agkistrodon contortrix	13	2	1	10
Agkistrodon piscivorus	39	8	10	21
Cemophora coccinea	28	5	5	18
Coluber constrictor	57	13	5	39
Coluber flagellum	25	4	7	14
Crotalus adamanteus	25	0	1	24
Crotalus horridus	15	8	1	6
Diadophis punctatus	11	0	0	11
Farancia abacura	27	11	1	15
Farancia erytrogramma	4	1	1	2
Haldea striatula	2	0	0	2
Heterodon platirhinos	35	13	4	18
Heterodon simus	3	0	0	3
Lampropeltis elapsoides	8	2	1	5
Lampropeltis getula	16	0	2	14
Liodytes alleni	4	2	0	2
Liodytes pygaea	15	2	2	11
Liodytes rigida	20	7	6	7
Micrurus fulvius	4	0	1	3
Nerodia erythrogaster	27	7	1	19
Nerodia fasciata	69	25	11	33
Nerodia floridana	2	1	0	1
Nerodia taxispilota	33	8	4	21
Opheodrys aestivus	19	1	2	16
Pantherophis alleghaniensis	61	10	15	36
Pantherophis guttatus	32	6	4	22
Pituophis melanoleucus	9	2	0	7
Sistrurus miliarius	16	1	2	13
Storeria dekayi	2	0	0	2
Storeria occipitomaculata	2	1	0	1
Thamnophis sauritus	19	4	5	10
Thamnophis sirtalis	36	6	9	21
Virginia valeriae	1	0	0	1
**TOTAL:**	**679**	**150**	**101**	**428**

Supplementary Table S2: Contingency table showing number of snakes sampled for ophidiomycosis by species and year in Georgia, USA.

Species	Number sampled	2016	2017	2018
Agkistrodon contortrix	13	0	10	3
Agkistrodon piscivorus	39	1	28	10
Cemophora coccinea	28	0	22	6
Coluber constrictor	57	2	44	11
Coluber flagellum	25	1	20	4
Crotalus adamanteus	25	3	20	2
Crotalus horridus	15	3	7	5
Diadophis punctatus	11	0	5	6
Farancia abacura	27	0	8	19
Farancia erytrogramma	4	0	0	4
Haldea striatula	2	0	1	1
Heterodon platirhinos	35	6	18	11
Heterodon simus	3	2	1	0
Lampropeltis elapsoides	8	0	3	5
Lampropeltis getula	16	0	4	12
Liodytes alleni	4	0	1	3
Liodytes pygaea	15	0	3	12
Liodytes rigida	20	0	4	16
Micrurus fulvius	4	0	2	2
Nerodia erythrogaster	27	0	20	7
Nerodia fasciata	69	0	46	23
Nerodia floridana	2	0	1	1
Nerodia taxispilota	33	0	25	8
Opheodrys aestivus	19	0	12	7
Pantherophis alleghaniensis	61	3	35	23
Pantherophis guttatus	32	2	19	11
Pituophis melanoleucus	9	0	6	3
Sistrurus miliarius	16	0	11	5
Storeria dekayi	2	0	1	1
Storeria occipitomaculata	2	0	0	2
Thamnophis sauritus	19	0	5	14
Thamnophis sirtalis	36	0	20	16
Virginia valeriae	1	0	0	1
**TOTAL:**	**679**	**23**	**402**	**254**

Supplementary Table S3: Contingency table showing number of snakes sampled for ophidiomycosis by species and month in Georgia, USA from 2016 to 2018.

Species	Number sampled	January	February	March	April	May	June	July	August	September	October	November	December
Agkistrodon contortrix	13	0	0	0	0	1	7	2	1	1	0	1	0
Agkistrodon piscivorus	39	0	0	1	11	6	4	4	4	4	4	1	0
Cemophora coccinea	28	0	0	1	2	8	6	9	2	0	0	0	0
Coluber constrictor	57	0	4	1	15	6	9	9	4	1	3	2	3
Coluber flagellum	25	2	1	4	2	7	0	1	3	1	1	1	2
Crotalus adamanteus	25	2	3	1	0	0	4	2	3	2	1	4	3
Crotalus horridus	15	0	0	0	1	1	2	4	3	4	0	0	0
Diadophis punctatus	11	0	1	0	1	0	3	2	0	0	3	1	0
Farancia abacura	27	0	0	1	7	6	5	5	2	1	0	0	0
Farancia erytrogramma	4	0	0	1	0	1	2	0	0	0	0	0	0
Haldea striatula	2	0	0	0	0	0	0	0	2	0	0	0	0
Heterodon platirhinos	35	0	2	2	9	3	2	2	1	7	6	0	1
Heterodon simus	3	0	0	0	0	0	0	0	0	0	3	0	0
Lampropeltis elapsoides	8	0	0	0	1	2	2	0	1	0	0	2	0
Lampropeltis getula	16	0	0	1	1	7	1	4	1	1	0	0	0
Liodytes alleni	4	0	0	0	1	0	0	2	0	1	0	0	0
Liodytes pygaea	15	0	0	0	2	4	2	3	2	1	1	0	0
Liodytes rigida	20	0	0	0	4	2	4	7	0	2	1	0	0
Micrurus fulvius	4	0	0	0	1	1	0	0	0	0	1	1	0
Nerodia erythrogaster	27	0	0	2	9	9	3	2	1	0	1	0	0
Nerodia fasciata	69	1	2	8	8	3	6	21	10	6	3	1	0
Nerodia floridana	2	0	0	0	0	0	0	2	0	0	0	0	0
Nerodia taxispilota	33	0	0	8	6	5	6	1	2	5	0	0	0
Opheodrys aestivus	19	0	0	0	0	1	5	4	2	2	2	3	0

Pantherophis alleghaniensis	61	0	3	1	13	10	13	5	6	6	1	3	0
Pantherophis guttatus	32	0	1	0	3	7	7	4	4	2	0	3	1
Pituophis melanoleucus	9	0	1	1	3	0	1	1	0	0	0	1	1
Sistrurus miliarius	16	0	0	0	0	0	1	4	8	3	0	0	0
Storeria dekayi	2	0	0	0	0	0	1	0	1	0	0	0	0
Storeria occipitomaculata	2	0	0	0	0	0	0	1	1	0	0	0	0
Thamnophis sauritus	19	0	0	0	6	1	5	4	2	1	0	1	0
Thamnophis sirtalis	36	0	0	0	1	5	7	8	6	3	3	3	0
Virginia valeriae	1	0	0	0	0	0	0	1	0	0	0	0	0
**TOTAL:**	**679**	**5**	**18**	**33**	**107**	**96**	**108**	**113**	**72**	**54**	**34**	**28**	**11**

Supplementary Table S4: Contingency table showing number of snakes sampled for ophidiomycosis by species and presence of skin lesions in Georgia, USA from 2016 to 2018.

		Skin lesions present	
**Species**	**Number sampled**	**No**	**Yes**
Agkistrodon contortrix	13	11	2
Agkistrodon piscivorus	39	26	13
Cemophora coccinea	28	25	3
Coluber constrictor	57	40	17
Coluber flagellum	25	15	10
Crotalus adamanteus	25	18	7
Crotalus horridus	15	14	1
Diadophis punctatus	11	8	3
Farancia abacura	27	15	12
Farancia erytrogramma	4	2	2
Haldea striatula	2	1	1
Heterodon platirhinos	35	30	5
Heterodon simus	3	2	1
Lampropeltis elapsoides	8	8	0
Lampropeltis getula	16	12	4
Liodytes alleni	4	3	1
Liodytes pygaea	15	14	1
Liodytes rigida	20	15	5
Micrurus fulvius	4	4	0
Nerodia erythrogaster	27	11	16
Nerodia fasciata	69	56	13
Nerodia floridana	2	1	1
Nerodia taxispilota	33	18	15
Opheodrys aestivus	19	10	9
Pantherophis alleghaniensis	61	37	24
Pantherophis guttatus	32	29	3
Pituophis melanoleucus	9	5	4
Sistrurus miliarius	16	14	2
Storeria dekayi	2	2	0
Storeria occipitomaculata	2	2	0
Thamnophis sauritus	19	14	5
Thamnophis sirtalis	36	29	7
Virginia valeriae	1	1	0
**TOTAL:**	**679**	**492**	**187**

Supplementary Table S5: Contingency table showing number of snakes sampled by species and qPCR result for *Ophidiomyces ophiodiicola* in Georgia, USA from 2016 to 2018.

		qPCR result	
**Species**	**Number sampled**	**Negative**	**Positive**
Agkistrodon contortrix	13	12	1
Agkistrodon piscivorus	39	28	11
Cemophora coccinea	28	27	1
Coluber constrictor	57	50	7
Coluber flagellum	25	25	0
Crotalus adamanteus	25	19	6
Crotalus horridus	15	13	2
Diadophis punctatus	11	10	1
Farancia abacura	27	22	5
Farancia erytrogramma	4	3	1
Haldea striatula	2	2	0
Heterodon platirhinos	35	34	1
Heterodon simus	3	3	0
Lampropeltis elapsoides	8	8	0
Lampropeltis getula	16	11	5
Liodytes alleni	4	4	0
Liodytes pygaea	15	15	0
Liodytes rigida	20	20	0
Micrurus fulvius	4	4	0
Nerodia erythrogaster	27	16	11
Nerodia fasciata	69	62	7
Nerodia floridana	2	2	0
Nerodia taxispilota	33	18	15
Opheodrys aestivus	19	18	1
Pantherophis alleghaniensis	61	53	8
Pantherophis guttatus	32	31	1
Pituophis melanoleucus	9	8	1
Sistrurus miliarius	16	15	1
Storeria dekayi	2	2	0
Storeria occipitomaculata	2	2	0
Thamnophis sauritus	19	18	1
Thamnophis sirtalis	36	33	3
Virginia valeriae	1	1	0
**TOTAL:**	**679**	**589**	**90**

Supplementary Table S6: Contingency table showing number of snakes sampled for ophidiomycosis by age class and year in Georgia, USA.

Age Class	Number sampled	2016	2017	2018
**Juvenile**	150	8	89	53
**Subadult**	101	2	59	40
**Adult**	428	13	254	161
**TOTAL:**	**679**	**23**	**402**	**254**

Supplementary Table S7: Contingency table showing number of snakes sampled for ophidiomycosis by age class and month in Georgia, USA from 2016 to 2018.

Age Class	Number sampled	January	February	March	April	May	June	July	August	September	October	November	December
**Juvenile**	150	0	1	9	25	9	19	29	11	21	12	11	3
**Subadult**	101	2	3	4	16	21	15	18	13	3	4	2	0
**Adult**	428	3	14	20	66	66	74	66	48	30	18	15	8
**TOTAL:**	**679**	**5**	**18**	**33**	**107**	**96**	**108**	**113**	**72**	**54**	**34**	**28**	**11**

Supplementary Table S8: Contingency table showing number of snakes sampled for ophidiomycosis by age class and presence of skin lesions in Georgia, USA from 2016 to 2018.

		Skin lesions present	
**Age Class**	**Number sampled**	**No**	**Yes**
**Juvenile**	150	126	24
**Subadult**	101	78	23
**Adult**	428	288	140
**TOTAL:**	**679**	**492**	**187**

Supplementary Table S9: Contingency table showing number of snakes sampled by age class and qPCR result for *Ophidiomyces ophiodiicola* in Georgia, USA from 2016 to 2018.

		qPCR result	
**Age Class**	**Number sampled**	**Negative**	**Positive**
**Juvenile**	150	144	6
**Subadult**	101	90	11
**Adult**	428	355	73
**TOTAL:**	**679**	**589**	**90**

Supplementary Table S10: Contingency table showing number of snakes sampled for ophidiomycosis by year and presence/absence of skin lesions in Georgia, USA from 2016 to 2018.

		Skin lesions present	
**Year**	**Number sampled**	**No**	**Yes**
**2016**	23	20	3
**2017**	402	305	97
**2018**	254	167	87
**TOTAL:**	**679**	**492**	**187**

Supplementary Table S11: Contingency table showing number of snakes sampled by month and qPCR result for *Ophidiomyces ophiodiicola* in Georgia, USA from 2016 to 2018.

		qPCR result	
**Month**	**Number sampled**	**Negative**	**Positive**
**January**	5	5	0
**February**	18	13	5
**March**	33	18	15
**April**	107	92	15
**May**	96	85	11
**June**	108	97	11
**July**	113	103	10
**August**	72	62	10
**September**	54	47	7
**October**	34	31	3
**November**	28	26	2
**December**	11	10	1
**TOTAL:**	**679**	**589**	**90**

Supplementary Table S12: Contingency table showing number of snakes sampled for ophidiomycosis by month and year in Georgia, USA.

Month	Number sampled	2016	2017	2018
**January**	5	0	5	0
**February**	18	0	9	9
**March**	33	0	25	8
**April**	107	0	44	63
**May**	96	0	43	53
**June**	108	0	62	46
**July**	113	0	54	59
**August**	72	0	56	16
**September**	54	13	41	0
**October**	34	3	31	0
**November**	28	3	25	0
**December**	11	4	7	0
**TOTAL:**	**679**	**23**	**402**	**254**

Supplementary Table S13: Contingency table showing number of snakes sampled by age class and ophidiomycosis category in Georgia, USA from 2016 to 2018.

		Ophidiomycosis Category			
**Age Class**	**Number sampled**	**Negative**	***Ophidiomyces* present**	**Possible ophidiomycosis**	**Apparent ophidiomycosis**
**Juvenile**	150	125	1	20	4
**Subadult**	101	74	4	16	7
**Adult**	428	273	15	81	59
**TOTAL:**	**679**	**472**	**20**	**117**	**70**

Supplementary Table S14: Contingency table showing number of snakes sampled by species and ophidiomycosis category in Georgia, USA from 2016 to 2018.

		Ophidiomycosis category			
**Species**	**Number sampled**	**Negative**	***Ophidiomyces* present**	**Possible ophidiomycosis**	**Apparent ophidiomycosis**
Agkistrodon contortrix	13	11	0	1	1
Agkistrodon piscivorus	39	23	3	5	8
Cemophora coccinea	28	25	0	2	1
Coluber constrictor	57	40	0	10	7
Coluber flagellum	25	15	0	10	0
Crotalus adamanteus	25	18	0	2	6
Crotalus horridus	15	12	2	1	0
Diadophis punctatus	11	8	0	2	1
Farancia abacura	27	14	0	10	4
Farancia erytrogramma	4	2	0	1	1
Haldea striatula	2	**1**	0	1	0
Heterodon platirhinos	35	31	0	4	1
Heterodon simus	3	2	0	1	0
Lampropeltis elapsoides	8	8	0	0	0
Lampropeltis getula	16	9	3	2	2
Liodytes alleni	4	3	0	1	0
Liodytes pygaea	15	13	0	1	0
Liodytes rigida	20	11	0	4	1
Micrurus fulvius	4	4	0	0	0
Nerodia erythrogaster	27	9	2	6	10
Nerodia fasciata	69	51	3	9	4
Nerodia floridana	2	1	0	1	0
Nerodia taxispilota	33	14	4	4	11
Opheodrys aestivus	19	10	0	8	1
Pantherophis alleghaniensis	61	36	1	21	7
Pantherophis guttatus	32	28	1	3	0
Pituophis melanoleucus	9	5	0	3	1
Sistrurus miliarius	16	14	0	1	1
Storeria dekayi	2	2	0	0	0
Storeria occipitomaculata	2	2	0	0	0
Thamnophis sauritus	19	9	0	4	1
Thamnophis sirtalis	36	27	1	5	2
Virginia valeriae	1	1	0	0	0
**TOTAL:**	**679**	**472**	**20**	**117**	**70**

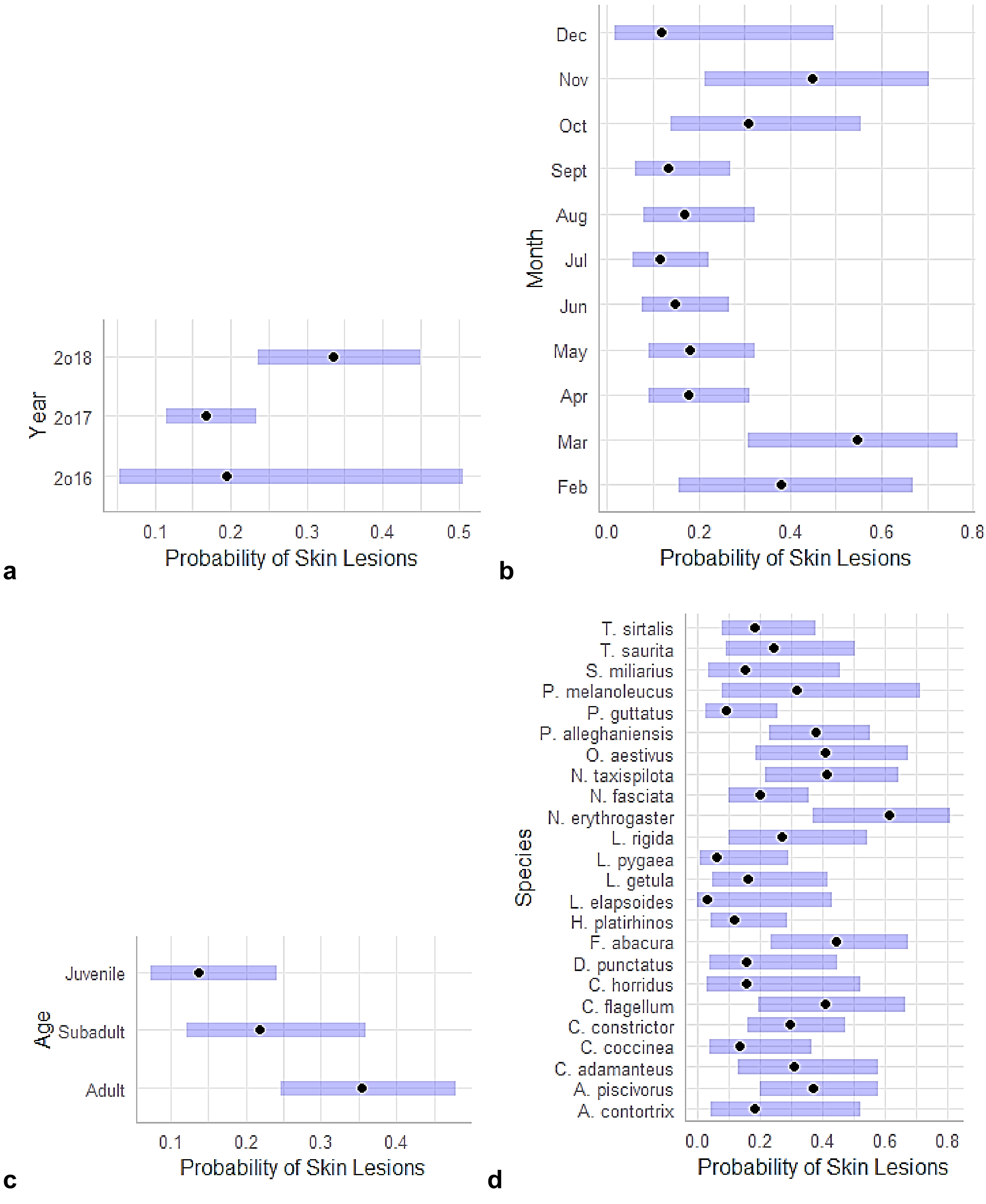

Supplementary Figure S1. Effect size plots (marginal mean values with 95% confidence intervals) estimated from a generalized linear model predicting the probability of skin lesion presence using the additive effects of year (**a**), month (**b**), age class (**c**), and species (**d**).

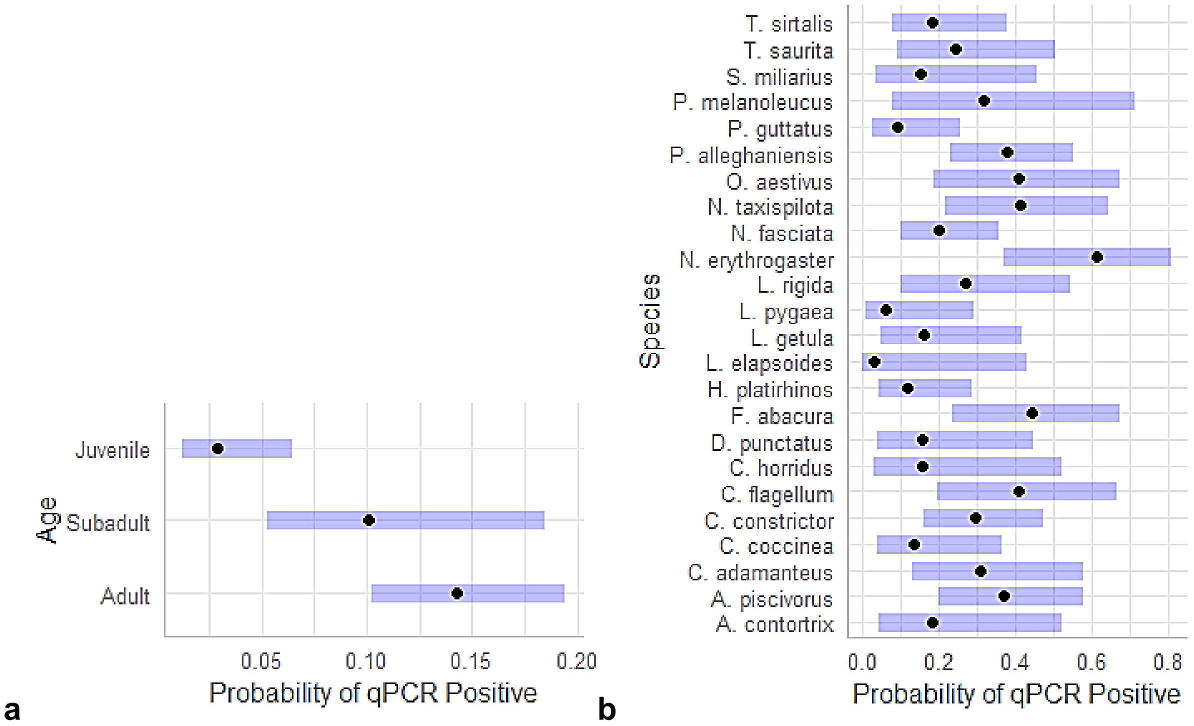

Supplementary Figure S2. Effect size plots (marginal mean values with 95% confidence intervals) estimated from a generalized linear model predicting the probability of testing qPCR positive for *Ophidiomyces ophiodiicola* using the additive effects of age class (**a**) and species (**b**).

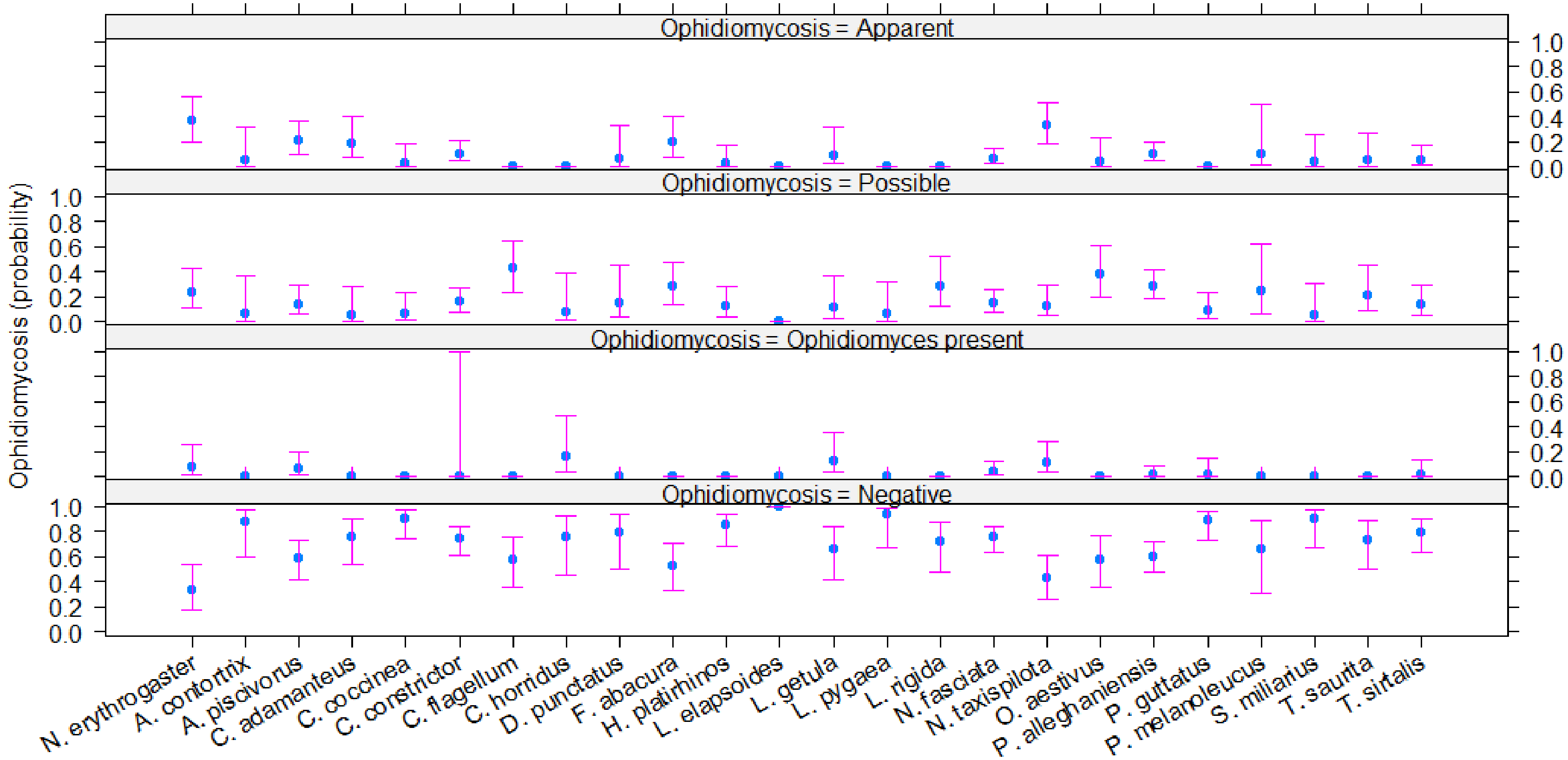

Supplementary Figure S3. Effect size plots (marginal mean values with 95% confidence intervals) estimated from a multinomial logistic regression model predicting ophidiomycosis category using the additive effects of age class and species. Eastern indigo snakes (*Drymarchon couperi*) are excluded.

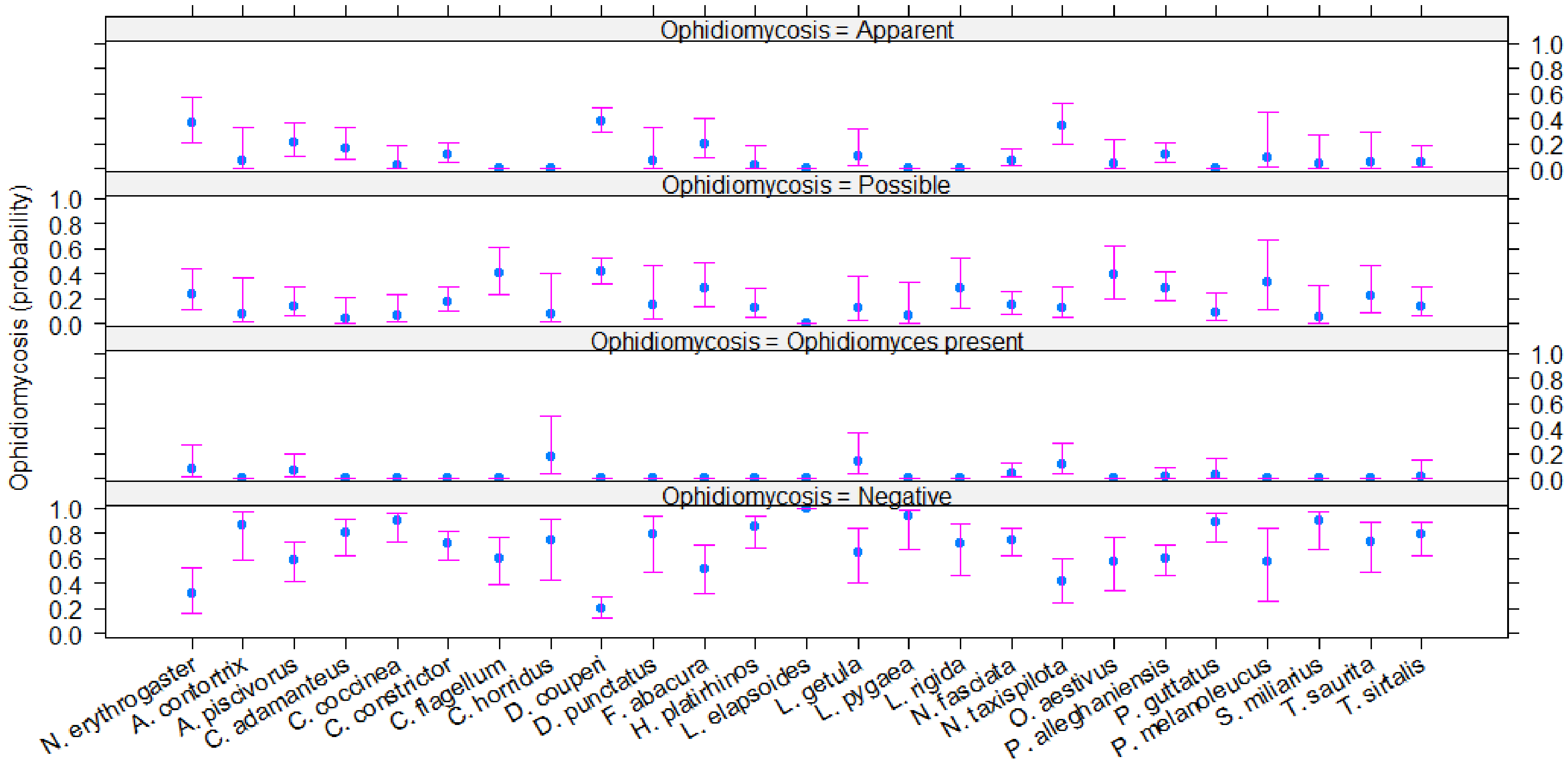

Supplementary Figure S4. Effect size plots (marginal mean values with 95% confidence intervals) estimated from a multinomial logistic regression model predicting ophidiomycosis category using the additive effects of age class and species. Eastern indigo snakes (*Drymarchon couperi*) are included.

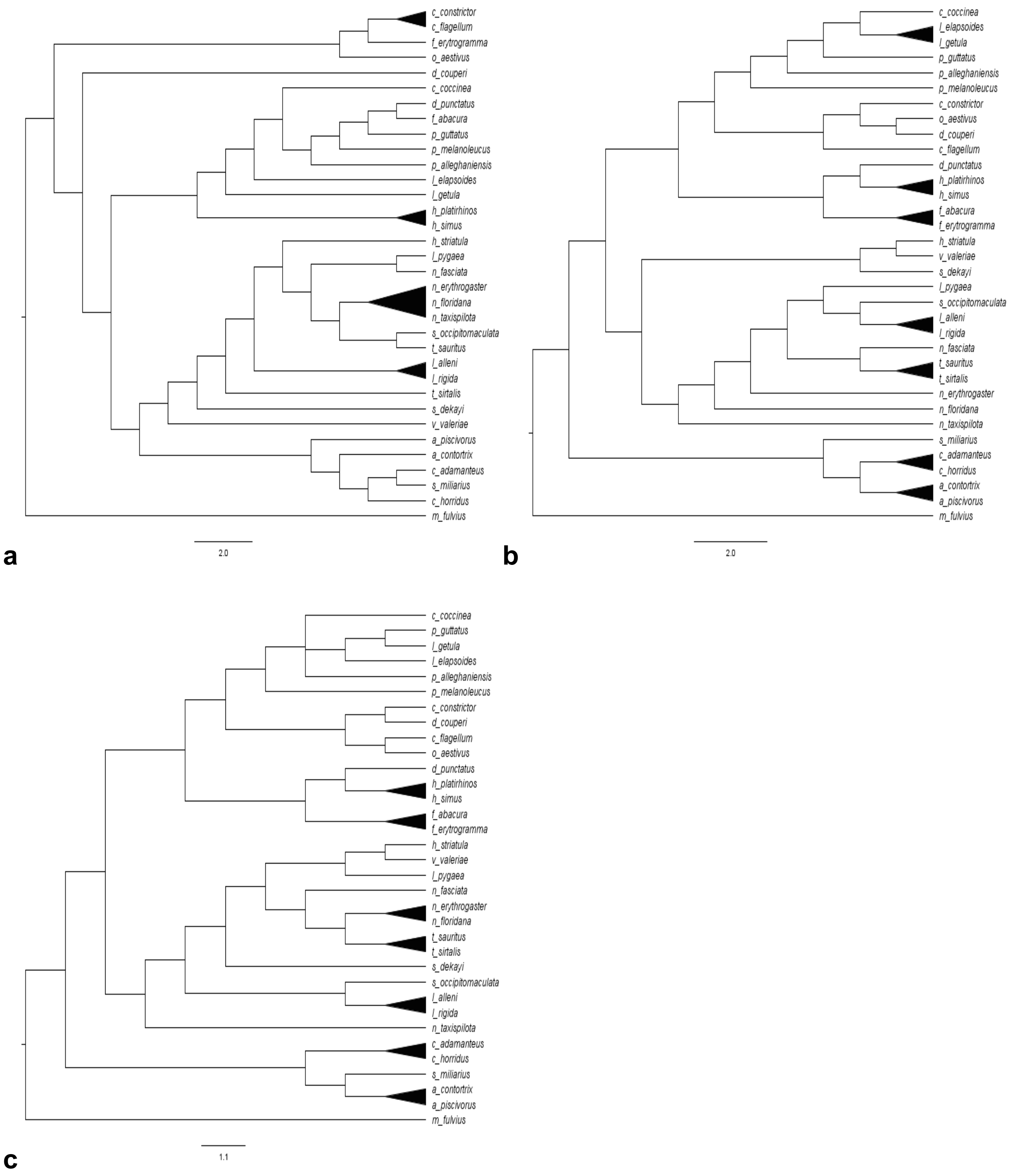

Supplemental Figure S5. Phylogenetic reconstruction from protein sequences using different algorithms: (**a**) Least squares distance algorithm; (**b**) Maximum parsimony algorithm; (**c**) Maximum likelihood algorithm. Triangles indicate species of the same genus grouped together on adjacent branches of the tree.

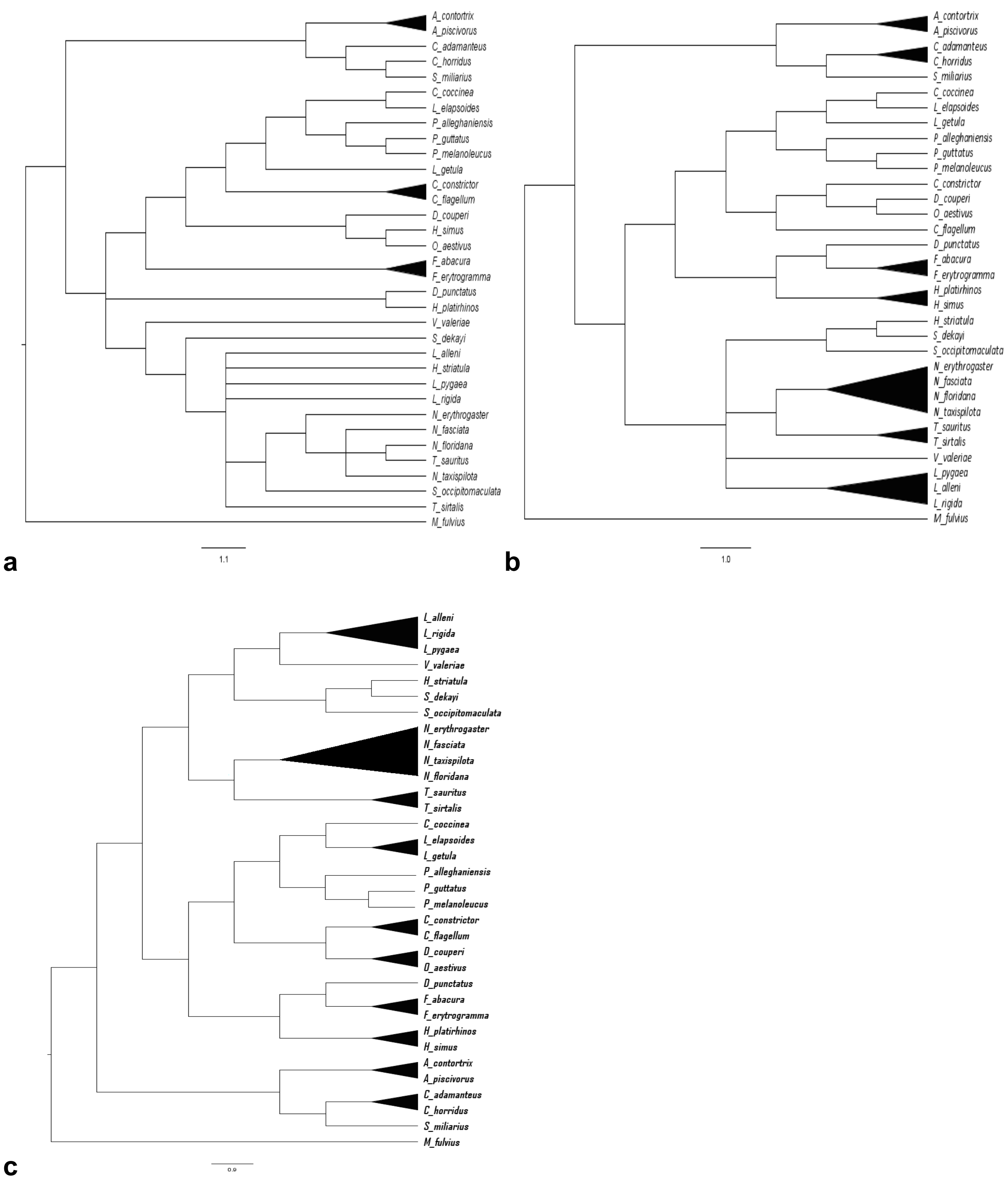

Supplemental Figure S6. Phylogenetic reconstruction from cDNA sequences using different algorithms: (**a**) Least squares distance algorithm; (**b**) Maximum parsimony algorithm; (**c**) Maximum likelihood algorithm. Triangles indicate species of the same genus grouped together on adjacent branches of the tree.
